# Dynamic Hormonal Networks in Flax During *Fusarium oxysporum* Infection and Their Regulation by Spermidine

**DOI:** 10.3390/molecules30234631

**Published:** 2025-12-02

**Authors:** Beata Augustyniak, Ivan Petrik, Danuse Tarkowska, Marta Burgberger, Wioleta Wojtasik, Ondrej Novak, Anna Kulma

**Affiliations:** 1 Faculty of Biotechnology, University of Wrocław, Przybyszewskiego 63/77, 51-148 Wroclaw, Poland; beata.augustyniak@uwr.edu.pl (B.A.); marta.burgberger@uwr.edu.pl (M.B.); wioleta.wojtasik@uwr.edu.pl (W.W.); 2 Laboratory of Growth Regulators, Institute of Experimental Botany of the Czech Academy of Sciences, Palacký University, Šlechtitelů 27, 783 71 Olomouc, Czech Republic; ivan.petrik@upol.cz (I.P.); danuse.tarkowska@upol.cz (D.T.); ondrej.novak@upol.cz (O.N.)

**Keywords:** flax, *Fusarium oxysporum*, spermidine, phytohormones, plant defence, hormonal crosstalk

## Abstract

**Background:** Flax (*Linum usitatissimum* L.) is an economically important crop that is highly susceptible to *Fusarium oxysporum* f. sp. *lini* (Foln). While phytohormones are key regulators of defence, their interaction with polyamines during infection remains poorly understood. This study aimed to characterise hormonal dynamics in flax under Foln infection and the modulatory role of spermidine (Spd). **Methods:** Targeted UPLC–MS/MS profiling quantified over 30 hormone-related compounds, including auxins, cytokinins, gibberellins, jasmonates, salicylic acid, and abscisic acid, in shoots and roots of healthy, infected, and Spd-treated plants. Two Spd concentrations (10 and 100 mM) were applied under controlled in vitro conditions. **Results:** Foln infection triggered tissue- and time-specific hormonal shifts, with early activation of jasmonate and auxin metabolism in shoots and later accumulation of salicylic acid and gibberellins in roots. Spd, particularly at 10 mM, reshaped these responses by reinforcing cytokinin and salicylic acid responses, stabilising auxin homeostasis, and enhancing jasmonate and abscisic acid responses. **Conclusions:** Spermidine coordinates hormone crosstalk, enabling balanced and efficient defence activation. The results highlight its potential as a priming agent enhancing flax resilience to *F. oxysporum*.

## 1. Introduction

Flax (*Linum usitatissimum* L.) is a temperate crop of considerable economic value, cultivated for both its oil-rich seeds and fibrous stems. Flaxseed oil, abundant in α-linolenic acid, is widely used in food, cosmetics, and industrial applications, while flax fibres are employed in textiles and composite materials. Moreover, the versatility of flax by-products such as seedcake and shives positions the crop as a promising candidate for zero-waste agriculture [1,2].

Despite its utility and sustainability, flax remains highly susceptible to soil-borne pathogens, most notably *Fusarium oxysporum* f. sp. *lini* (Foln), which causes fusarium wilt and leads to severe yield and quality losses [3]. Foln penetrates the plant through the root system and colonises vascular tissues, disrupting water and nutrient transport. This results in characteristic symptoms such as chlorosis, wilting, and ultimately plant death [4]. Its ability to persist in soil makes management particularly difficult, highlighting the need for sustainable strategies to strengthen the plant’s innate immunity.

Plant responses to pathogen attack are orchestrated by phytohormones—small signalling molecules that regulate both development and defence. Salicylic acid (SA), jasmonic acid (JA), and abscisic acid (ABA) are central in activating immune pathways, including systemic acquired resistance (SAR) and stomatal closure [5]. Growth-related hormones such as auxins (AUX), cytokinins (CKs), and gibberellins (GAs) are also closely linked to defence, undergoing redistribution or metabolic conversion during infection [6]. These compounds exist in multiple active, conjugated, or precursor forms, which enable precise and tissue-specific regulation of plant responses [7].

Alongside hormones, polyamines (PAs) such as putrescine (Put), spermidine (Spd), and spermine (Spm) are known to influence defence. They can act as signalling molecules, induce PR gene expression, stimulate mitogen-activated protein kinase (MAPK) cascades, and contribute to the reinforcement of structural barriers [8,9]. Importantly, elevated PA levels may either promote resistance or increase susceptibility, depending on the host–pathogen system. For example, Spm treatment has been found to enhance resistance in *Arabidopsis thaliana* and *Solanum lycopersicum* against *Botrytis cinerea* through reactive oxygen species (ROS) generation and SAR signalling [10], whereas higher Spd levels in tomato have been shown to increase susceptibility [11].

Our previous work showed that Foln infection in flax induces dynamic changes in PA metabolism, accompanied by ROS bursts and activation of defence-related genes such as chitinase. We also demonstrated that exogenous Spd can enhance resistance, reducing fungal colonisation while promoting a controlled, transient oxidative response and balanced defence activation [12]. These findings suggest that Spd may act as a regulatory molecule, fine-tuning immunity in a redox-dependent manner.

However, while the involvement of PAs in defence is increasingly recognised, their interaction with phytohormone signalling under pathogen attack remains largely unexplored. Transcriptomic analyses of flax infected with Foln revealed strong induction of genes responsive to SA, JA, ethylene (ET), and ABA, indicating the activation of a complex defence network. SA-regulated transcripts dominated the early response in the resistant cultivar, together with elevated WRKY transcription factors, whereas the susceptible cultivar exhibited a prolonged ET-associated response. These findings suggest that Foln resistance in flax results from the coordinated action of SA, JA, ET and ABA signalling pathways, which may interact with polyamine metabolism during infection [13].

Here, we present a comprehensive analysis of hormonal changes in flax during Foln infection, quantifying over 30 hormone-related compounds—including active forms and biosynthetic intermediates of IAA, CKs, GAs, JA, SA, and ABA—in roots and shoots of healthy and infected plants. This dataset provides a unique, tissue-specific view of the hormonal landscape shaped by fungal stress and represents, to our knowledge, the first study of this kind in flax–pathogen interactions. Building on this, we further investigate how exogenous Spd modulates these processes, integrating targeted hormone profiling with previously published data on PA metabolism, gene expression, and fungal colonisation. Our goal is to clarify whether Spd functions merely as a stress modifier or acts as a true priming agent capable of reshaping hormonal crosstalk and enhancing flax resilience under biotic stress.

## 2. Results

### 2.1. Progression of the Infection with Fusarium oxysporum in Flax

In summary, the pathogen colonised roots more strongly than shoots, with fungal DNA increasing progressively over time and accompanied by a delayed but measurable spread to the aerial parts. Molecular defence responses followed a similar pattern: chitinase transcript levels rose significantly in roots and later in shoots, and infection progression was associated with tissue-specific changes in polyamine metabolism and redox status, including a transient H_2_O_2_ burst and induction of key PA biosynthetic genes. Those changes are reported in detail in our earlier publication by Augustyniak et al. (2025) [12]. These findings provided the physiological framework for the current hormone-focused analysis and confirmed the suitability of this plant material for studying hormonal regulation during Fusarium infection.

### 2.2. Hormonal Changes in Flax Plants Infected with Fusarium oxysporum

**Figure 1:**
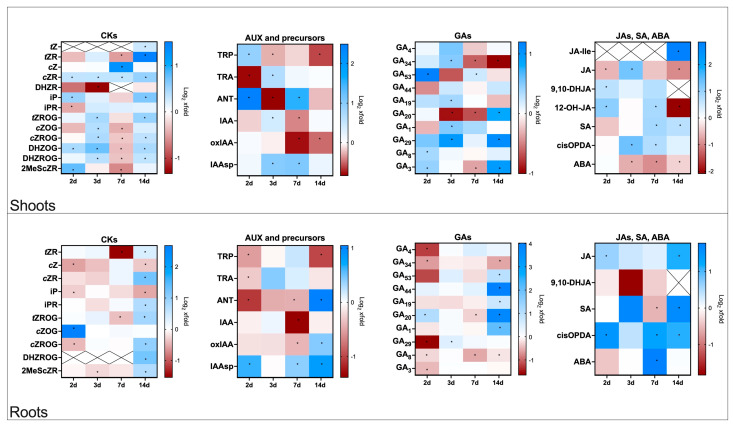
Phytohormone content in flax shoots and roots after *F. oxysporum* application. Heat map box represents the mean from three replicates. The significance of differences between groups was determined using two-way ANOVA followed by Tukey’ s post hoc test (* *p* < 0.05 for comparison to control from the same time point as the sample). This figure was made using GraphPad Prism 10 software. CKs—cytokinins, *t*Z—*trans*-zeatin, *t*ZR—*trans*-zeatin riboside, *c*Z—*cis*-zeatin, *c*ZR—*cis*-zeatin riboside, DHZR—dihydrozeatin riboside, iP—N^6^—isopentenyladenine, iPR—N^6^—isopentenyladenosine, *t*ZROG—*trans*-zeatin riboside-*O*-glucoside, *c*ZOG—*cis*-zeatin-*O*-glucoside, *c*ZROG—*cis*-zeatin riboside *O*-glucoside, DHZOG—dihydrozeatin-*O*-glucoside, DHZROG—dihydrozeatin riboside-*O*-glucoside, 2MeScZR—2-methylthio-*cis*-zeatin riboside, AUX—auxins, TRP—tryptophan, TRA—tryptamine, ANT—anthranilic acid, IAA—indole-3-acetic acid, oxIAA—2-oxindole-3-acetic acid, IAAsp—indole-3-acetyl-aspartate, Gas—gibberellins, JAs—jasmonates, JA-Ile—jasmonoyl-isoleucine, JA—jasmonic acid, 9,10-DHJA—9,10-dihydrojasmonic acid, 12-OH-JA—12-hydroxy-jasmonic acid, *cis*-OPDA—12-oxo-phytodienoic acid, SA—salicylic acid, ABA—abscisic acid.

The data presented below show changes in hormone levels in flax plants infected with Foln. Values are expressed as fold changes relative to noninfected control plants. Time points correspond to 2-, 3-, 7-, and 14-days post Foln application (dpa). The results are shown in Figure 1, with corresponding absolute concentrations (pmol/g fresh weight) listed in Supplementary Tables.

#### 2.2.1. Cytokinin Profiles in Roots and Shoots of Flax Plants Infected with *F. oxysporum*

Foln infection induces a transient activation of cytokinin signalling in the shoots, while the roots undergo an early and more sustained depletion of bioactive CK forms. The progressive enrichment of riboside and O-glucosylated CKs, particularly in roots at later stages, points to enhanced turnover and inactivation rather than simple accumulation, suggesting an active reprogramming of CK homeostasis under pathogen stress (Figure 1).

As measured, the most abundant CKs in roots are *O*-glucosylated derivatives of *cis*-zeatin (*c*ZOG ~10 pmol/g, *c*ZROG ~4 pmol/g), followed by free-base forms such as *c*Z (~0.7 pmol/g) and iP (~0.7 pmol/g), and the ribosides *c*ZR (~0.4 pmol/g) and iPR (~0.3 pmol/g). The levels of the most bioactive *trans*-zeatin (*t*Z) and its riboside (*t*ZR) were low or below the detection limit. The most abundant CK forms in shoots were *c*ZOG (~8 pmol/g) and *c*ZROG (~9 pmol/g), accompanied by measurable amounts of *c*Z (~0.1 pmol/g), iP (~0.08 pmol/g), and *t*ZR (~0.09 pmol/g). Riboside levels (*c*ZR, iPR) were comparable to those in roots. Average values from all time points studied are given in parentheses. The exact values are given in Tables S1 and S2.

Shoots exhibited transient accumulation of active forms. A moderate increase (2.5-fold) in *c*Z was observed 7 days after Foln application, while both *t*Z and iP levels were elevated at day 14 (1.2-fold and 1.6-fold, respectively). iP also showed a minor increase (1.4-fold) 2 days after Foln application. Among riboside forms, *c*ZR displayed steady accumulation throughout the infection period. *t*ZR underwent a 0.7-fold decrease 7 days after Foln application but increased again by day 14 (2.8-fold). iPR levels dropped by 30% 2 days after Foln application but returned to control values later. Dihydrozeatin riboside (DHZR) decreased early and remained low. *O*-glucosylated cytokinins transiently increased at days 2 and 3, dipped at day 7, and rose again at day 14. The profile of methylthio-*c*ZR (2MeS*c*ZR) resembled that of the conjugated forms (Figure 1).

In the roots, a reduction in CK active forms (*c*Z, iP, and their ribosides) was observed as early as 2 days after Foln application. This trend continued or intensified by day 7. By day 14, *c*Z and iP remained suppressed (20% decrease and 30% decrease, respectively), while the levels of *c*ZR and iPR showed a moderate increase (2.4-fold and 1.7-fold, respectively). Notably, the accumulation of *O*-glucosylated CKs intensified at this late stage, particularly *c*ZROG (2.0-fold). *t*ZR decreased significantly (70% decrease) 7 days after Foln application and partially recovered by day 14. 2MeS*c*ZR followed a similar pattern to other ribosides, with increased levels at the final time point (Figure 1).

#### 2.2.2. Auxin Profiles in Roots and Shoots of Flax Plants Infected with *F. oxysporum*

Under non-infected conditions, auxin-related metabolites showed distinct tissue-specific distributions. In the shoots, the dominant compound was tryptophan (TRP, ~108,377 pmol/g), the primary precursor of auxin biosynthesis. Roots accumulated significantly lower levels of TRP (~29,821 pmol/g) and had a slightly lower concentration of inactive degradation products oxIAA (~5 pmol/g) and aspartate conjugate IAAsp (~0.8 pmol/g) compared to shoots (~10 pmol/g and ~1.5 pmol/g, respectively). IAA itself was present at a similar concentration in both shoots (~10 pmol/g) and roots (~13 pmol/g). Average values from all time points studied are given in parentheses. The exact values are given in Tables S3 and S4.

In the shoots, a 1.9-fold increase in TRP was observed 2 days after Foln application, followed by a gradual reduction. TRA (tryptamine) displayed an inverse pattern, decreasing by 40% at day 2 and increasing at day 3 (1.6-fold). The levels of ANT (anthranilic acid), auxin biosynthetic precursor in the TRP-dependent biosynthetic pathway were highly dynamic, with strong peaks 2 (5.6-fold) and 7 (3.0-fold) days after Foln application and an intermediate drop at day 3 (40% decrease), reflecting possible fluctuations in the tryptophan pathway. The most bioactive auxin IAA increased modestly (1.3-fold) 3 days after Foln application, followed by a 20% decline at day 7. Notably, oxIAA levels decreased significantly at later time points (days 7 and 14, 40% decrease and 30% decrease, respectively), while IAAsp showed a marked increase at days 3 and 7 (2.0-fold and 2.1-fold) (Figure 1).

In the roots, auxin-related changes were generally delayed and more subdued. TRP and TRA levels decreased 2 days after Foln application (30% decrease and 20% decrease, respectively), with no major recovery in later stages. ANT was reduced early but showed a late 2-fold increase 14 days after Foln application. IAA remained largely unchanged except for a 60% decrease 7 days after Foln application, mirroring the timing observed in shoots. oxIAA levels decreased moderately 7 days after Foln application (20% decrease), then increased 14 days after Foln application (1.3-fold), while IAAsp consistently increased (Figure 1).

Overall, these patterns suggest transient, finely tuned activation of the tryptophan-dependent auxin pathway in shoots, leading to a modest, short-lived rise in IAA followed by enhanced conjugation (IAAsp) and reduced oxidative catabolism (oxIAA), indicative of active auxin turnover rather than sustained accumulation. In roots, the weaker and delayed changes in TRP pathway intermediates and IAA, alongside a steady increase in IAAsp, point to a more conservative adjustment of auxin homeostasis.

#### 2.2.3. Gibberellin Profiles in Roots and Shoots of Flax Plants Infected with *F. oxysporum*

Under control conditions, total GA content was higher in the roots than in the shoots. Notably, the root tissue accumulated markedly higher levels of biologically inactive catabolites GA_34_ (~0.08 pmol/g in roots, ~0.01 pmol/g in shoots) and GA_29_ (~0.3 pmol/g in roots and ~0.04 pmol/g in shoots), while the shoot tissue exhibited a broader distribution of biosynthetic precursors, including GA_19_ (~0.3 pmol/g in shoots and ~0.1 pmol/g in roots). Bioactive gibberellins (GA_1_, GA_3_, GA_4_) constituted a greater proportion of the total gibberellin pool in the shoots, although both tissues maintained relatively low levels of these forms overall. The abundance of their precursors (GA_53_, GA_44_, GA_19_, GA_20_) was greater in the shoots, while their catabolites (GA_8_, GA_34_) were more prevalent in roots, accounting for over 70% of the total GA pool in this tissue. Average values from all time points studied are given in parentheses. The exact values are given in Tables S5 and S6.

The infection triggered only modest and transient changes in gibberellin GA levels in the shoots. Early accumulation (2.3-fold) of the precursor GA_53_ was detected 2 days after Foln application but was not sustained. GA_1_ and GA_3_ showed moderate, short-lived increases 3 days after Foln application and 2 and 14 days after Foln application, respectively. Notably, the level of GA_34_, the 2-hydroxy catabolite of GA_4_, increased (1.4-fold) 3 days after Foln application, followed by a steady decline at later stages of cultivation. Conversely, levels of most other precursors (GA_19_, GA_20_) and active form GA_4_ remained relatively stable, with no consistent or statistically significant infection-related patterns. The shoot tissue exhibited a relatively buffered hormonal response, with transient metabolic shifts limited to early or late infection stages (Figure 1).

In the roots, GA dynamics were more pronounced and temporally structured. A transient reduction in GA_4_ (50% decrease) and its catabolite GA_34_ (40% decrease) was observed 2 days after Foln application. However, 14 days after Foln application, several precursors (GA_20_, GA_19_, GA_53_, GA_44_) and the active form GA_1_ accumulated strongly, particularly GA_20_ and GA_44_, which showed over tenfold increases. Meanwhile, GA_8_ and GA_3_ levels remained suppressed throughout the infection period, reinforcing the notion of a selective shift in gibberellin metabolism (Figure 1).

The gradual reduction in active GA forms points to growth suppression consistent with defence prioritisation over biomass accumulation.

#### 2.2.4. Profiles of Jasmonates, Salicylic Acid, and Abscisic Acid in Flax Plants Infected with *F. oxysporum*

In shoots, the most abundant compound was *cis*-OPDA (~1469.7 pmol/g), a bioactive biosynthetic precursor of JA, followed by 12-OH-JA (12-hydroxyjasmonic acid; ~4 pmol/g), SA (~2 pmol/g) and ABA (~1.5 pmol/g). Similarly, in roots, *cis*-OPDA levels were the highest (~677.9 pmol/g), while SA (~8 pmol/g) and ABA (~0.3 pmol/g) were moderately accumulated. Average values from all time points studied are given in parentheses. The exact values are given in Tables S7 and S8.

In the shoots, the JA pathway was transiently activated. JA levels dropped by 30% 2 days after Foln application but peaked at day 3 (2.5-fold), followed by a 50% decline. Its derivatives, 9,10-DHJA (9,10-dihydrojasmonic acid) and 12-OH-JA, showed elevated levels early (1.6-fold and 2.2-fold, respectively, 2 days after Foln application), with 12-OH-JA also increasing 7 days after Foln application (1.8-fold), before sharply decreasing by day 14 (80% decrease). The bioactive JA isoleucine conjugate (JA-Ile) remained undetectable until day 14, when a strong increase compared to the control (7.2-fold) was observed. In parallel, SA increased at later time points, while *cis*-OPDA rose 3 and 7 days after Foln application. In contrast, ABA levels progressively declined 3 days after Foln application (Figure 1).

In the roots, jasmonate-related responses were more sustained. JA levels increased significantly 2 (1.5-fold) and 14 (2.2-fold) days after Foln application, and *cis*-OPDA was elevated throughout most time points, reflecting enhanced biosynthesis. While 9,10-DHJA remained relatively stable and JA-Ile was undetectable, ABA exhibited a sharp increase (3.5-fold) only 7 days after Foln application. SA showed a late induction, increasing (3.1-fold) significantly 14 days after Foln application (Figure 1).

The delayed JA-Ile and SA accumulation indicates sequential activation of jasmonate- and salicylate-mediated defence pathways, while transient ABA elevation reflects stress adaptation mechanisms.

### 2.3. Hormonal Changes in Spermidine-Treated, Non-Infected Flax Plants

**Figure 2:**
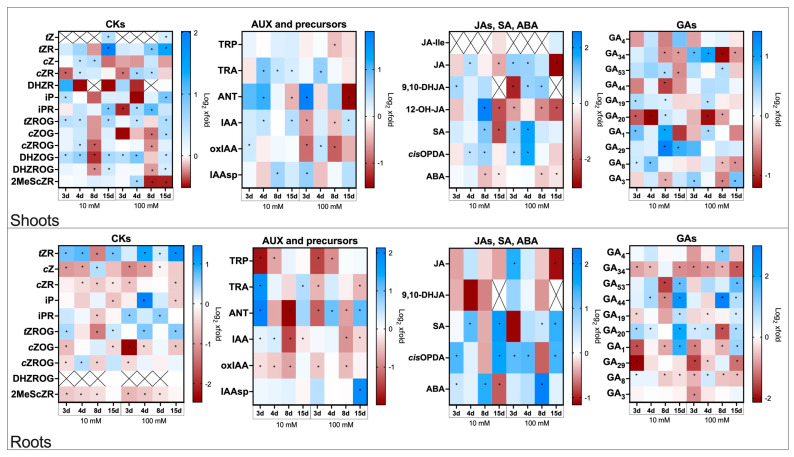
Phytohormones content in flax shoots and roots after Spd treatment. Heat map box represents the mean from three replicates. The significance of differences between groups was determined using two-way ANOVA followed by Tukey’s post hoc test (* *p* < 0.05 for comparison to control from the same time point as the sample). This figure was made using GraphPad Prism 10 software. CKs—cytokinins, *t*Z—*trans*-zeatin, *t*ZR—*trans*-zeatin riboside, *c*Z—*cis*-zeatin, *c*ZR—*cis*-zeatin riboside, DHZR—dihydrozeatin riboside, iP—N^6^—isopentenyladenine, iPR—N^6^—isopentenyladenosine, *t*ZROG—*trans*-zeatin riboside-*O*-glucoside, *c*ZOG—*cis*-zeatin-*O*-glucoside, *c*ZROG—*cis*-zeatin riboside *O*-glucoside, DHZOG—dihydrozeatin-*O*-glucoside, DHZROG—dihydrozeatin riboside-*O*-glucoside, 2MeScZR—2-methylthio-*cis*-zeatin riboside, AUX—auxins, TRP—tryptophan, TRA—tryptamine, ANT—anthranilic acid, IAA—indole-3-acetic acid, oxIAA—2-oxindole-3-acetic acid, IAAsp—indole-3-acetyl-aspartate, Gas—gibberellins, JAs—jasmonates, JA-Ile—jasmonoyl-isoleucine, JA—jasmonic acid, 9,10-DHJA—9,10-dihydrojasmonic acid, 12-OH-JA—12-hydroxy-jasmonic acid, *cis*-OPDA—12-oxo-phytodienoic acid, SA—salicylic acid, ABA—abscisic acid.

The data presented below reflect hormone level changes in flax plants treated with Spd in the absence of infection. Values are expressed as fold changes relative to untreated, non-infected controls. Time points are given as days after Spd treatment (3, 4, 8, and 15 days). The results are shown in Figure 2, with corresponding absolute concentrations (pmol/g FW) provided in Supplementary Tables.

#### 2.3.1. Cytokinins Profiles in Roots and Shoots of Flax Plants Treated with Spd

Spermidine treatment modulated cytokinin pools, potentially priming developmental and metabolic processes without inducing stress-like signatures (Figure 2).

In shoots, *t*Z was undetectable at early time points but exhibited a moderate increase 15 days after Spd treatment, reaching 1.4-fold and 1.2-fold increases compared to control for 10 mM and 100 mM Spd, respectively. A more pronounced and delayed response was observed for *t*ZR, which remained unchanged until day 15 in the 10 mM treatment in which an increase was observed, while 100 mM Spd induced earlier upregulation 8 days after Spd treatment (1.8-fold) and further elevation at day 15 (2.8-fold).

Changes in *c*Z and *c*ZR were transient. The 10 mM Spd treatment elevated *c*Z 4 and 8 days after Spd treatment (1.3-fold and 1.5-fold), while *c*ZR showed a 20% decrease at day 3, followed by a modest increase at day 4 (1.3-fold). The 100 mM treatment produced a similar pattern for *c*ZR.

Among isopentenyl-type CKs, iP increased significantly at day 3 in shoots of plants treated with 10 mM Spd (1.4-fold), while a delayed increase was noted at day 15 with 100 mM Spd (1.9-fold). iPR also showed late accumulation (1.8-fold at day 15) in the 10 mM treatment, whereas the 100 mM dose induced a 20% decrease at day 3 followed by increases at days 4 (1.5-fold) and 8 (2.1-fold).

Among conjugated forms, *t*ZROG and DHZOG displayed consistent upregulation, particularly at day 15, while *c*ZOG and *c*ZROG exhibited weaker and variable changes. Notably, DHZOG showed a dynamic pattern with increases at early and late time points and a transient drop at day 8. In contrast, DHZR remained unaffected.

Lastly, the methylated CK 2MeScZR was unaffected by 10 mM Spd treatment, whereas treatment with 100 mM Spd induced a transient increase at day 4 (1.5-fold), followed by consistent 30% decreases at days 8 and 15.

In roots, *t*ZR levels increased consistently across time points in both treatments, especially at 100 mM Spd (up to 2.5-fold at day 15). In contrast, *c*Z, *c*ZR, *c*ZOG, and 2MeScZR were generally downregulated, particularly between days 3 and 8 (for *c*Z and *c*ZR reductions ranging from 20% to 60% in both treatments; *c*ZOG consistently declined in both treatments notably by 80% at day 3 for 100 mM SPD; 2MeScZR reduction ranged from 20% to 40%). Some glucosylated forms (*t*ZROG, *c*ZROG) showed transient upregulation, while DHZROG, which was undetectable at early stages, accumulated moderately at day 15 after treatment with both Spd concentrations.

#### 2.3.2. Auxin Profiles in Roots and Shoots of Flax Plants Treated with Spd

Altered auxin precursor levels under Spd treatment suggest a polyamine-dependent adjustment of auxin biosynthesis and turnover during growth regulation (Figure 2).

In shoots, 10 mM Spd treatment generally promoted auxin accumulation, with consistent increases observed for TRA, ANT, and IAA at multiple time points. In contrast, the 100 mM Spd treatment induced more transient and sometimes opposing effects, including early increases (e.g., ANT 3.6-fold at day 3) followed by pronounced declines at later stages (e.g., ANT 0.3-fold at day 15).

Oxidative and conjugated auxin forms also exhibited dose-dependent patterns. For oxIAA after 10 mM Spd treatment, a mild increase was observed at day 3 (1.2-fold), while 100 mM Spd caused a sharp decline at days 3 and 8 (50% decrease), with a transient increase at day 4 (1.3-fold). For the conjugated derivative IAAsp, 10 mM Spd induced a delayed increase at day 8 (1.5-fold), and 100 mM Spd led to an earlier upregulation at day 3 (1.6-fold).

In roots, auxin metabolism appeared more sensitive to Spd. Early time points showed a strong and consistent decline in TRP and oxIAA regardless of dose. However, active forms such as TRA and ANT were stimulated by 10 mM Spd (with strong increases at day 3), while the same compounds were variably regulated or even suppressed after 100 mM Spd treatment. IAA tended to accumulate at early time points after 10 mM Spd but declined later, while the 100 mM dose predominantly caused decreases. Notably, IAAsp increased sharply at day 15 in roots only after 100 mM Spd treatment.

#### 2.3.3. Jasmonates, Salicylic Acid, and Abscisic Acid Profiles in Roots and Shoots of Flax Plants Treated with Spd

Spermidine caused only moderate changes in stress-related hormones, supporting the notion that Spd does not act as a stressor but may fine-tune signalling networks (Figure 2).

In shoots, JA and its derivatives (especially 12-OH-JA) were transiently elevated, with early increases followed by strong declines, particularly after 100 mM Spd. While 10 mM Spd triggered the accumulation of 12-OH-JA at day 8, 100 mM Spd led to consistent repression of this compound. The level of *cis*-OPDA was increased under both treatments, though increases were earlier and stronger at the higher dose. Root jasmonate responses were weaker, with only JA responding significantly to 100 mM Spd treatment (increased at day 3 (2.8-fold) and decreased by 50% at day 15)

SA also responded in a dose- and tissue-specific manner. In shoots, 100 mM Spd treatment induced strong and early SA accumulation, while 10 mM caused a delayed increase followed by a decrease. In roots, SA levels increased at later time points after both treatments, with stronger effects after 10 mM Spd.

ABA showed a consistent downward trend in shoots, with reduced levels at days 8 and 15, regardless of Spd dose. In contrast, root ABA levels rose markedly under both treatments, with a peak after 100 mM Spd at day 8 (5.1-fold), indicating tissue-specific divergence in ABA regulation following Spd treatment.

#### 2.3.4. Gibberellin Profiles in Roots and Shoots of Flax Plants Treated with Spd

In shoots, 10 mM Spd tended to promote the accumulation of bioactive gibberellin GA_1_, its biosynthetic precursor GA_19_, and catabolite GA_29_ at later time points, whereas 100 mM SPD often triggered early increases followed by suppression, especially for catabolites GA_34_ and GA_8_. Notably, GA_1_ and GA_29_ showed strong accumulation in response to 10 mM Spd, while GA_34_, GA_8_, and bioactive GA_3_ were reduced later in the time course, particularly after high-dose treatment.

In roots, gibberellin changes were more pronounced. In the biosynthetic branch of 13-hydroxylated GAs GA_1_, GA_20_, GA_44_, and GA_53_ were markedly elevated at day 15 after both Spd doses, with stronger effects at 10 mM. Most of the detected inactive GA catabolites (GA_34_, GA_29_, and GA_8_) were consistently lowly abundant, especially under 100 mM Spd.

Reduced GA levels in Spd-treated plants may indicate a polyamine-driven shift toward controlled growth and resource allocation.

### 2.4. Hormonal Changes in Spermidine-Treated Flax Plants Infected with Fusarium oxysporum

**Figure 3:**
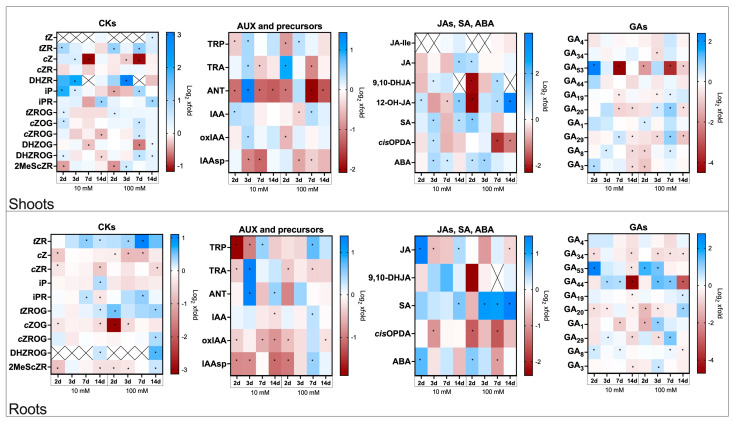
Phytohormones content in flax shoots and roots after Spd treatment and *F. oxysporum* application. Heat map box represents the mean from three replicates. The significance of differences between groups was determined using two-way ANOVA followed by Tukey’ s post hoc test (* *p* < 0.05 for comparison to control from the same time point as the sample). This figure was made using GraphPad Prism 10 software. CKs—cytokinins, *t*Z—*trans*-zeatin, *t*ZR—*trans*-zeatin riboside, *c*Z—*cis*-zeatin, *c*ZR—*cis*-zeatin riboside, DHZR—dihydrozeatin riboside, iP—N^6^—isopentenyladenine, iPR—N^6^—isopentenyladenosine, *t*ZROG—*trans*-zeatin riboside-*O*-glucoside, *c*ZOG—*cis*-zeatin-*O*-glucoside, *c*ZROG—*cis*-zeatin riboside *O*-glucoside, DHZOG—dihydrozeatin-*O*-glucoside, DHZROG—dihydrozeatin riboside-*O*-glucoside, 2MeScZR—2-methylthio-*cis*-zeatin riboside, AUX—auxins, TRP—tryptophan, TRA—tryptamine, ANT—anthranilic acid, IAA—indole-3-acetic acid, oxIAA—2-oxindole-3-acetic acid, IAAsp—indole-3-acetyl-aspartate, Gas—gibberellins, JAs—jasmonates, JA-Ile—jasmonoyl-isoleucine, JA—jasmonic acid, 9,10-DHJA—9,10-dihydrojasmonic acid, 12-OH-JA—12-hydroxy-jasmonic acid, *cis*-OPDA—12-oxo-phytodienoic acid, SA—salicylic acid, ABA—abscisic acid.

The data presented below show hormone level changes in flax plants treated with Spd and infected with Foln. Values are expressed as fold changes relative to Spd-untreated, Foln-infected controls. Time points correspond to 2-, 3-, 7-, and 14 dpa. The results are shown in Figure 3, with corresponding absolute concentrations (pmol/g fresh weight) listed in Supplementary Tables.

#### 2.4.1. Cytokinins Profiles in Roots and Shoots in Spermidine-Treated Flax Plants Infected with *Fusarium oxysporum*

In Spd-treated, infected plants, cytokinin dynamics were dampened compared to infection-only conditions, suggesting mitigation of stress-induced hormonal imbalance (Figure 3).

Spd treatment in infected plants led to early and pronounced increases in *t*ZR and iP levels, particularly after 100 mM Spd treatment. Notably, *t*ZR and iP peaked within 2–3 dpa while their conjugates (iPR) showed delayed accumulation at day 14. A strong but transient rise in DHZR was observed, especially under 100 mM Spd.

In contrast, *c*Z and *c*ZR were mostly downregulated in both tissues, especially the roots, suggesting reduced synthesis or increased degradation under stress. Glycosylated derivatives such as *t*ZROG and *c*ZOG showed mild, time-specific increases, while DHZOG and DHZROG displayed mixed trends—early downregulation followed by moderate recovery.

Roots showed delayed but consistent increases in *t*ZR levels. In contrast, *c*Z was reduced early, especially after 100 mM Spd treatment, with significant declines at days 2, 3, and 7 by 20%, 40% and 50%, respectively. *c*ZR also decreased 2 and 14 dpa after 10 mM Spd treatment (20% decrease and 40% decrease, respectively), and 14 dpa after 100 mM Spd treatment (30% decrease).

*i*P was elevated only after 10 mM Spd treatment at day 14 (1.2-fold), while iPR showed a transient increase at day 7 (1.2- and 1.3-fold for 10 and 100 mM Spd, respectively), followed by a 20% decline at day 14 after 10 mM Spd.

Among glucosylated forms, *t*ZROG was upregulated at day 14 after both treatments (10 mM: 1.1-fold; 100 mM: 1.3-fold), with an earlier rise at day 2 under 100 mM Spd (1.4-fold). *c*ZOG decreased in most conditions, with the most prominent reduction seen at day 2 after 100 mM Spd (90% decrease). *c*ZROG increased only at day 14 after 100 mM Spd (1.2-fold), with no significant changes after 10 mM.

DHZROG increased at 14 dpa after both treatments (1.2-fold for 10 mM, 1.5-fold for 100 mM). After both treatments, 2MeScZR levels decreased at early time points, especially under 10 mM Spd (40% decrease at day 2) but rebounded slightly at 14 dpa after 100 mM Spd (1.2-fold).

#### 2.4.2. Auxin Profiles in Roots and Shoots in Spermidine-Treated Flax Plants Infected with *Fusarium oxysporum*

Spd application moderated auxin turnover during infection, preventing excessive hormonal disruption associated with pathogen-triggered tissue remodeling (Figure 3).

In the shoots, early and transient increases in auxin precursors (TRP, TRA) and ANT were observed, particularly 2–3 dpa. Notably, ANT was strongly induced by 10 mM Spd but suppressed at 100 mM. Free IAA showed modest and inconsistent fluctuations, while oxIAA and IAAsp generally decreased.

In the roots, there was a stronger tendency toward decreased levels of both precursors and deactivation products, especially under 10 mM Spd. Temporary increases in ANT and TRA levels occurred at 3–7 dpa. The level of oxIAA was consistently reduced under 10 mM Spd at all time-points (20% to 40% decrease) and showed similar downward trends after 100 mM Spd at days 2 and 14 (30% decrease and 10% decrease). IAAsp dropped significantly under both treatments, especially under 10 mM Spd (50% decrease), while a late increase at day 7 was observed only under 100 mM Spd (1.4-fold).

#### 2.4.3. Jasmonates, Salicylic Acid and Abscisic Acid Profiles in Roots and Shoots in Spermidine-Treated Flax Plants Infected with *Fusarium oxysporum*

Spermidine modified the stress-hormone signature by reducing the amplitude or delaying jasmonate and salicylate peaks, supporting its role as a defence modulator that prevents the overactivation of stress responses (Figure 3).

In the shoots, JA, 9,10-DHJA and 12-OH-JA generally increased following Spd treatment, with the most pronounced effect observed for 12-OH-JA at 14 dpa after 100 mM Spd (13.4-fold). In the roots, free JA content increased significantly after 10 mM Spd at 2 and 14 dpa (2.8-fold and 1.3-fold) but decreased by 30% at 3 dpa. In contrast, 100 mM Spd led to a reduction in JA levels at 14 dpa (20% decrease). No significant changes were noted in 9,10-DHJA levels under either treatment.

SA accumulation in the shoots was stimulated by both Spd concentrations, although 100 mM Spd triggered an earlier response (2 dpa), while 10 mM Spd caused increases at later timepoints. A similar trend was observed in the roots. Shoot levels of *cis*-OPDA showed opposing trends depending on the concentration, increasing after 10 mM Spd but decreasing after 100 mM. In the roots, *cis*-OPDA levels dropped at 3 dpi after 10 mM Spd treatment (50% decrease), and even more strongly under 100 mM SPD, with reductions at 2 and 7 dpa (70% decrease in both cases).

ABA content also increased in response to Spd, especially in early timepoints. In shoots, the rise was earlier and stronger with 100 mM Spd. In roots, ABA peaked at 2 dpa after both concentrations, followed by a decline under the higher dose.

#### 2.4.4. Gibberellin Profiles in Roots and Shoots in Spermidine-Treated Flax Plants Infected with *Fusarium oxysporum*

GA levels were partially restored relative to infected plants, indicating that spermidine alleviates infection-driven growth suppression (Figure 3).

In shoots, inactive precursor GA_53_ displayed the strongest fluctuation, with a sharp induction after 10 mM Spd (2 dpa 5.2-fold) and drastic reduction at 7 dpa, while 100 mM led to consistent decreases. Another inactive precursor GA_20_ and catabolite GA_29_ were also responsive, showing increases at earlier timepoints followed by declines at later stages. Bioactive GA_1_ and GA_3_ as well as catabolite GA_8_ showed moderate stimulation at early timepoints, especially at 100 mM, but tended to drop by day 14. The levels of bioactive GA_4_ and inactive precursor GA_44_ remained unchanged.

In roots, GA_53_ and GA_44_ were strongly upregulated by both Spd concentrations, especially during early infection stages. Notably, GA_53_ peaked after 10 mM Spd (2 dpa 6.9-fold) and after 100 mM Spd (3 dpa 2.5-fold), while GA_44_ reached up to 2.7-fold. GA_20_, GA_1_, and GA_29_ also showed concentration-dependent modulation, including increases at later stages. In contrast, GA_34_ consistently decreased across timepoints and treatments, particularly after 100 mM Spd.

## 3. Discussion

The analysis of phytohormone profiles in flax (*Linum usitatissimum*) following infection with *Fusarium oxysporum* provides valuable insights into the plant’s defence responses and hormonal regulation under biotic stress. The hormonal landscape of flax plants subjected to *F. oxysporum* infection revealed a dynamic, tissue-specific response involving growth-regulating and defence-associated hormones. Our results demonstrate significant alterations in the levels of key plant hormones, suggesting that the hormonal signalling network plays a central role in mediating flax responses to *Fusarium* infection, in turn regulating metabolic plant response.

Temporal profiling indicated that jasmonates were among the earliest responders, followed by salicylic acid, abscisic acid, and later shifts in gibberellin and cytokinin levels. This progression is consistent with a biphasic stress response, wherein early perception of the pathogen triggers rapid defence activation, followed by longer-term modulation of growth and resource allocation.

The observed increase in SA and JA levels in infected tissues is consistent with their well-documented roles in plant immunity. SA is typically associated with SAR and is effective against biotrophic pathogens, while JA is linked to induced systemic resistance (ISR) and defence against necrotrophs [14]. Given that *F. oxysporum* is considered a hemibiotrophic pathogen [15], the simultaneous activation of both pathways may reflect a complex and dynamic defence strategy employed by flax.

In the shoots, early jasmonate accumulation—particularly of JA and its oxidised derivatives—was observed during Foln infection while the bioactive JA-Ile became detectable only 14 days after Foln application. This indicates a tightly regulated jasmonate burst, a phenomenon previously described as critical for early defence activation against *F. oxysporum* in *Arabidopsis* and banana plants [16,17]. Interestingly, increased jasmonate levels in flax appeared to be transient, aligning with the notion that prolonged activation may be detrimental to energy balance or immune coordination [18].

At later stages, SA accumulated progressively in both tissues, particularly in roots 14 days after Foln application, potentially marking the onset of SAR. This SA elevation in roots was accompanied by decreased ABA levels in shoots and a transient ABA peak in roots, consistent with prior findings suggesting antagonistic or context-dependent roles of these hormones during infection [19,20]. Consistent with this, our earlier work demonstrated that *F. oxysporum* infection in flax activates the plastidial terpenoid pathway, resulting in elevated ABA accumulation and induction of numerous PR genes, including chitinases, β-1,3-glucanases, and thaumatin-like proteins [21]. This suggests that ABA contributes positively to the early defence response, but that its increased levels must remain transient, as prolonged signalling would likely impose significant costs on growth and energy balance. Such coordinated hormonal shifts suggest a division of labour between root and shoot tissues, with roots engaging in sustained defence signalling, while shoots temporarily suppress growth-related hormonal activity.

Auxin and cytokinin responses further exemplify this spatial divergence. In shoots, a transient rise in IAA and active cytokinins (*c*Z, iP) occurred at early days after Foln application, followed by an increase in their conjugated or inactive forms (IAAsp, *c*ZROG). Similar patterns were delayed or absent in roots, with most hormonal changes emerging after day 7, suggesting a more gradual or controlled adjustment in below-ground tissues. This aligns with models proposed by Di et al. [22], who highlighted that Foln–host interactions are strongly influenced by shifts in the phytohormonal network. They pointed out that auxin signalling and transport, in particular, contribute both to initial colonisation of roots and to the manifestation of shoot disease symptoms, while interactions with other hormone pathways such as SA and JA can further modulate resistance or susceptibility. From this perspective, the transient auxin and cytokinin fluctuations we observed may represent one component of a broader regulatory framework shaping the plant response to Foln.

Gibberellins, particularly their precursors and catabolites, exhibited prominent late-stage increases in roots but remained largely unaltered in shoots. This could indicate a potential regenerative or compensatory growth mechanism activated locally in infected root tissue. Notably, both GA_1_ and GA_20_ increased significantly 14 days after Foln application in roots, supporting the idea of de novo biosynthesis rather than simple redistribution. These findings align with the transcriptomic patterns reported by Galindo-González & Deyholos [23], who observed the induction of hormone-related genes in flax roots during *F. oxysporum* infection. In addition, in the transgenic flax line exhibiting higher resistance to *Fusarium* infection, a slight increase in GA_3_ levels was observed. Together with the data presented here, this may suggest a potential role of bioactive gibberellins in the plant defence response [24].

The temporal dynamics of hormonal responses observed in this study align with the molecular and phenotypic markers of infection progression. The onset of visible disease symptoms by the fourth week after Foln application corresponded with a strong and gradual accumulation of fungal DNA, particularly in roots, where the DNA amount of the *murein transglycosylase* gene increased more than 180-fold by day 14. This was paralleled by a substantial induction of *chitinase* transcripts. Notably, these changes coincided with tissue-specific hormonal shifts. The early rise in JA levels—especially in shoots—preceded the significant increase in *chitinase* expression and may underline this hormone’s importance in regulating the expression of defence-related genes. The subsequent increase in SA and ABA, particularly in roots, matched the later stages of infection, where both fungal load and *chitinase* transcript accumulation peaked [12]. This suggests that hormonal changes not only precede but also track the intensification of the infection, supporting a model in which hormone-mediated defence and growth regulation are tightly coupled to pathogen progression in a time- and tissue-dependent manner.

Taken together, the hormonal responses of flax plants to *F. oxysporum* appear to be both spatially and temporally stratified: jasmonate- and auxin-related mechanisms dominate the early phase of infection in shoots, while salicylic acid and gibberellin responses become more pronounced in roots during the later phase. Depending on the tissue and time point, ABA may play both activating and suppressive roles. These results underscore the complexity of hormonal crosstalk in biotic stress responses and provide a detailed physiological context for interpreting defence strategies in flax.

Analysis of flax hormonal profiles revealed that Spd application substantially modified the plant’s response to *Fusarium oxysporum* infection, affecting both hormone intensity and timing. While some general trends were preserved, Spd clearly modulated the amplitude, duration, and direction of many of these changes.

Spd treatment enhanced changes in several CK levels when compared to infection on its own. Additionally, Spd promoted the accumulation of conjugated forms, indicating a shift toward hormone stabilisation and possibly fine-tuned signalling. CKs are known to modulate both SA- and JA-dependent defences and can act as priming signals in biotic stress responses [25], suggesting that the observed enhancement in cytokinin accumulation after Spd application may facilitate more coordinated defence activation.

Spd treatment moderated a sharp fluctuation in auxin metabolites (observed in infected roots and infected plants). Notably, IAA levels became more stable, and decreases in oxIAA were less pronounced, indicating improved auxin balance under stress. Combined treatment also led to temporally coordinated increases in TRP and TRA, implying altered auxin biosynthesis or signalling dynamics. Crosstalk between auxin and PAs has been described in various contexts [26], with PAs capable of modulating auxin transport and conjugation, potentially explaining the stabilisation of auxin homeostasis observed here.

While JA-related compounds showed a dynamic response to infection alone, Spd treatment accelerated their accumulation and increased their magnitude, particularly 12-OH-JA and JA at later time points. SA peaks also occurred earlier and were more pronounced. SA–JA antagonism and its regulation by PAs is well-documented. Earlier reports demonstrated that elevated Spm levels can trigger gene expression of defence-related and JA- and MeJa-related genes [27] and can lead to increased JA levels [28]. Interestingly, studies on *A. thaliana* have shown that Spm deficiency shifts the balance between JA and SA responses by stimulating JA biosynthesis and reducing SA responses, leading to increased resistance to *B. cinerea* [29]. In our system, 10 mM Spd appeared to produce a balanced SA–JA response, whereas 100 mM Spd triggered stronger early SA peaks, potentially reflecting a more stress-intense signalling environment.

Similarly, ABA levels were boosted by Spd at early stages, though this effect waned over time. PAs are known to influence ABA biosynthesis and perception, often through redox-mediated pathways [30], suggesting that early ABA elevation in our study may be linked to Spd-induced H_2_O_2_ signalling. Indeed, PA oxidation produces H_2_O_2_, which acts as both a direct antimicrobial and a signalling molecule modulating hormone networks [31].

In response to infection alone, several GAs declined, while active forms such as GA_1_ and GA_3_ remained largely unchanged. Spd altered this pattern by promoting temporary increases in both biosynthetic intermediates and active GAs, particularly GA_3_ in shoots and GA_53_ in roots after 10 mM Spd treatment. Maintaining active GA pools under pathogen stress may help balance growth and defence—a trade-off commonly observed in plant immunity [32].

In addition to its effects under pathogen challenge, Spd application alone induced distinct hormonal and metabolic shifts, indicating that Spd may function as a direct signalling molecule capable of reprogramming plant metabolism in the absence of stress. Spd treatment without infection led to moderate but consistent increases in several CKs, as well as early rises in ABA, especially at 10 mM. These changes occurred without concomitant changes in jasmonates or and auxin catabolites, suggesting that Spd selectively targets hormone pathways linked to growth regulation and early defence readiness. Similar priming-like hormone responses to PAs have been observed in other species. In wheat, exogenous Spd and Spm applied during grain filling increased CK levels (zeatin and zeatin riboside) as well as ABA under non-stress conditions [33]. In *A. thaliana*, exogenous Spm, as well as elevated endogenous Spm in transgenic lines, enhanced ABA accumulation and induced the expression of ABA-responsive genes even in the absence of stress [34].

Our earlier analysis of the same plant material [12] revealed that Spd alone also modifies the endogenous PA pool, increasing not only Spd itself but also Put and, in some cases, Spm. Notably, these PA shifts were accompanied by modest but measurable increases in H_2_O_2_ levels even in the absence of infection, suggesting that PA oxidation was initiated as a signalling event. Given that H_2_O_2_ is a potent modulator of ABA and CK signalling [31], it is plausible that the hormone changes observed here resulted at least in part from Spd-induced redox signalling.

We reported previously that exogenous Spd significantly altered the endogenous PA pool and triggered a transient burst of H_2_O_2_ two days after *F. oxysporum* application [12], followed by restoration of redox homeostasis at later stages. This oxidative burst coincides with the earliest increases in SA and ABA reported in this manuscript. Gene expression analysis showed that Spd treatment modulated the expression of PA catabolic genes (*DAO*, *PAO*) [12], which are directly responsible for H_2_O_2_ generation, and *chitinase*, a canonical JA/ET-dependent defence marker. At 10 mM Spd, *DAO* and *PAO* showed moderate induction accompanied by a transient rise in *chitinase* transcripts, consistent with controlled ROS production and balanced JA–SA levels. In contrast, 100 mM Spd caused stronger and more sustained upregulation of both *oxidase* genes and *chitinase*, in line with the higher and earlier SA peaks observed in the hormonal data. Together, these results suggest that the extent of Spd-induced ROS production and subsequent hormonal crosstalk determines the intensity of downstream defence gene activation, with 10 mM Spd supporting an efficient but metabolically economical defence. In comparison, 100 mM Spd drives a more energetically demanding response, which may ultimately be detrimental to plant survival.

## 4. Conclusions and Prospects

This study shows that *Fusarium oxysporum* infection reshapes the hormonal landscape of flax, affecting cytokinin balance, auxin turnover, gibberellin levels, and jasmonate–salicylate defence pathways. Spermidine application modulated these responses in infected plants, leading to a faster, stronger, and in many cases, more balanced hormonal response. This included the following: of CK and SA responses, stabilisation of auxin metabolism, enhanced and timely activation of JA/ABA, and recovery of active GAs.

Such changes may underlie the observed improvements in phenotypic condition and reduced pathogen content in Spd-treated plants, especially at the 10 mM concentration. These findings align with the concept of polyamines acting as modulators of hormone networks during biotic stress and suggest that spermidine can enhance plant resilience by coordinating hormone-driven defence mechanisms. This regulatory role is consistent with previous findings in other systems, where exogenous PAs enhanced resistance to various fungal pathogens, including *Alternaria alternata* and *Fusarium graminearum* [35,36].

Future work should clarify the regulatory points at which polyamines interact with hormone pathways and verify the observed effects under soil-grown conditions and across different cultivars.

## 5. Materials and Methods

### 5.1. Plant Material and Growing Conditions

Seeds of flax (*Linum usitatissimum* L., cv. Nike) were provided by the Flax and Hemp Collection of the Institute of Natural Fibres and Medicinal Plants—National Research Centre (INF\&MP-NRC, Poznań, Poland). The pathogenic fungus *Fusarium oxysporum* f. sp. *lini* (Bolley) Snyder et Hansen (ATCC MYA-1201) was obtained from the American Type Culture Collection (ATCC, Manassas, VA, USA).

Seeds were surface-sterilised through immersion in 50% PPM solution (Plant Cell Technology, Washington, DC, USA) for 10 min, then transferred into sterile culture jars containing 45 mL of Murashige and Skoog (MS) medium (Sigma-Aldrich, St. Louis, MO, USA) supplemented with 10 g of vermiculite and adjusted to pH 5.8. Plants were maintained under controlled conditions in a growth chamber (16 h light at 21 °C/8 h dark at 16 °C).

At day 12 of in vitro culture, plants were treated with a solution of spermidine. Two concentrations were applied: 10 mM and 100 mM Spd (Sigma-Aldrich, St. Louis, MO, USA). A sterile 1 M stock solution of spermidine was prepared and diluted with sterile water to obtain 10 mM and 100 mM working solutions. One day after Spd treatment, half of the jars were inoculated with *F. oxysporum*, while the others served as non-infected controls.

For inoculation, fungal cultures were grown on potato dextrose agar (PDA, IBI Scientific, Dubuque, IA, USA) at 28 °C. Spores were harvested by flooding plates with sterile water, and the suspension was adjusted to 10^6^ spores/mL using a hemocytometer. A volume of 0.5 mL of the suspension was added per jar. For control plants, 0.5 mL of sterile water was applied.

Plants (roots and shoots separately) were collected at 2, 3, 7, and 14 days after fungal application. Each sample represented pooled material from one jar containing 16 plants. Samples were frozen in liquid nitrogen, ground to a fine consistency, and stored at −80 °C until analyses.

To confirm fungal colonisation, the abundance of the *F. oxysporum murein transglycosylase* gene was quantified. The expression of defence-related genes (e.g., chitinase) was as reported in Augustyniak et al. (2025) [12].

### 5.2. Phytohormone Extraction and Quantification, Except Gibberellins

Phytohormones and related metabolites were extracted and analysed according to the protocol of Šimura et al. (2018) [37], with minor modifications. Approximately 15 mg of fresh shoot or root tissue was homogenised in a MM400 vibration mill (Retsch, Haan, Germany) with 1 mL of ice-cold 50% acetonitrile (*v*/*v*), containing a mixture of stable isotope-labelled internal standards (0.2 pmol of CK bases, 0.5 pmol of CK *O*-glucosides and nucleotides, 25 pmol of [^2^H_4_]-TRA, [^2^H_5_]-TRP, [^2^H_4_]-IAN, 5 pmol of [^2^H_4_]-ANT, [^2^H_5_]-IAM, 2.5 pmol of [^2^H_2_]-JA-Ile, 0.25 pmol of [^2^H_5_]-*cis*OPDA, 2.5 pmol of [^2^H_6_]-ABA; OlChemIm, Olomouc, Czech Republic). After 5 min sonication, the extraction was performed at 4 °C for 30 min using a Stuart SB3 benchtop laboratory rotator (Bibby Scientific, Staffordshire, UK). After centrifugation 36,670× *g* and 4 °C for 20 min), supernatants were subjected to solid-phase extraction (SPE) using Oasis HLB cartridges (30 mg/1 mL, Waters, Milford, MA, USA), preconditioned with methanol, and equilibrated with 50% acetonitrile. Flow-through and elution fractions (30% acetonitrile) were combined, evaporated in vacuo, and reconstituted in 40 µL of 30% acetonitrile before LC-MS/MS analysis.

Quantification was carried out using an Acquity UPLC I-Class system (Waters, Milford, MA, USA) coupled with a Xevo TQ-S triple quadrupole mass spectrometer and equipped with a CSH C18 column (150 × 2.1 mm, 1.7 µm). Separation was performed at 50 °C with a binary gradient of 0.01% formic acid in water and acetonitrile. Data were acquired in both positive and negative ESI modes, and hormone levels were determined using the isotope dilution method [38].

### 5.3. Gibberellin Extraction and Quantification

Gibberellin profiling was performed according to the protocol described by Urbanová et al. (2013) [39]. In brief, 15 mg of frozen plant tissue was homogenised to a fine consistency using 2.8 mm ceria stabilised zirconium oxide beads (Next Advance Inc., Troy, NY, USA) and a MM 400 vibration mill at a frequency of 27 Hz for 3 min (Retsch GmbH & Co. KG, Haan, Germany) with 1 mL of ice-cold 80% acetonitrile containing 5% formic acid, along with internal standards (deuterium-labelled gibberellins, 2 pmol each [^2^H_2_]GA_1_, [^2^H_2_]GA_4_, [^2^H_2_]GA_9_, [^2^H_2_]GA_19_, [^2^H_2_]GA_20_, [^2^H_2_]GA_24_, [^2^H_2_]GA_29_, [^2^H_2_]GA_34_ and [^2^H_2_]GA_44_; OlChemIm, Olomouc, Czech Republic). Extraction proceeded overnight at 4 °C and 17 rpm using a benchtop laboratory rotator Stuart SB3 (Bibby Scientific, Staffordshire, UK). The homogenates were centrifugated (36,670× *g*, 10 min, 4 °C) and the pelet was re-extracted for 1 h to maximise GA recovery. Combined extracts were evaporated to dryness in vacuo (Acid-Resistant Concentrator Labconco, Kansas City, MO, USA) and purified using mixed-modeOasis MAX cartridges (60 mg/3 mL, Waters, Wexford, Ireland). GAs were eluted with 0.2 M formic acid in acetonitrile, evaporated to dryness, and the sample was then reconstituted in mobile phase for UHPLC-MS/MS analysis.

Chromatographic separation of GAs was carried out using an Acquity I-Class system (Waters, Milford, MA, USA) with an Acquity UPLC® CSH™ C18 column (2.1 × 50 mm, 1.7 µm; Waters, Wexford, Ireland). After separation, GAs were detected in multiple-reaction monitoring (MRM) mode with an Xevo TQ-XS triple-stage quadrupole instrument (Waters, Milford, MA, USA) operating in negative ESI mode. Data acquisition was performed with MassLynx 4.2 software (Waters, Milford, MA, USA), and GA levels were quantified using the isotope dilution method [38].

### 5.4. Statistical Analysis

Each experimental group consisted of three jars, each containing 16 plants. Plants from a single jar were pooled into one biological replicate. Results are presented as the mean ± standard deviation (SD) of three biological replicates. Statistical analyses were carried out using GraphPad Prism 10 software. Significance between treatments was tested with two-way ANOVA followed by Tukey’s post hoc test.

## Data Availability

The raw data supporting the conclusions of this article will be made available by the authors on request.

## Supplementary Materials

The following supporting information can be downloaded at https://www.mdpi.com/article/10.3390/molecules30234631/s1: Table S1. Cytokinin content in roots of non-infected control flax plants and plants infected with *F. oxysporum*. Table S2. Cytokinin content in shoots of non-infected control flax plants and plants infected with *F. oxysporum*. Table S3. Auxin and precursor content in roots of non-infected control flax plants and plants infected with *F. oxysporum*. Table S4. Auxin and precursor content in shoots of non-infected control flax plants and plants infected with *F. oxysporum*. Table S5. Gibberellin content in roots of non-infected control flax plants and plants infected with *F. oxysporum*. Table S6. Gibberellin content in shoots of non-infected control flax plants and plants infected with *F.oxysporum*. Table S7. Jasmonate, salicylic acid, and abscisic content in roots of non-infected control flax plants and plants infected with *F.oxysporum*. Table S8. Jasmonate, salicylic acid, and abscisic content in shoots of non-infected control flax plants and plants infected with *F.oxysporum*. Table S9. Cytokinin content in roots of non-infected control flax plants and plants treated with Spd (10 mM and 100 mM). Table S10. Cytokinin content in shoots of non-infected control flax plants and plants treated with Spd (10 mM and 100 mM). Table S11. Auxin and precursor content in roots of non-infected control flax plants and plants treated with Spd (10 mM and 100 mM). Table S12. Auxin and precursor content in shoots of non-infected control flax plants and plants treated with Spd (10 mM and 100 mM). Table S13. Gibberellin content in roots of non-infected control flax plants and plants treated with Spd (10 mM and 100 mM). Table S14. Gibberellin content in shoots of non-infected control flax plants and plants treated with Spd (10 mM and 100 mM). Table S15. Jasmonate, salicylic acid, and abscisic content in roots of non-infected control flax plants and plants treated with Spd (10 mM and 100 mM). Table S16. Jasmonate, salicylic acid, and abscisic content in shoots of non-infected control flax plants and plants treated with Spd (10 mM and 100 mM). Table S17. Cytokinin content in roots of infected control flax plants and plants treated with Spd (10 mM and 100 mM) and infected with *F. oxysporum*. Table S18. Cytokinin content in shoots of infected control flax plants and plants treated with Spd (10 mM and 100 mM) and infected with *F. oxysporum*. Table S19. Auxin and precursor content in roots of infected control flax plants and plants treated with Spd (10 mM and 100 mM) and infected with *F. oxysporum*. Table S20. Auxin and precursor content in shoots of infected control flax plants and plants treated with Spd (10 mM and 100 mM) and infected with *F. oxysporum*. Table S21. Gibberelin content in roots of infected control flax plants and plants treated with Spd (10 mM and 100 mM) and infected with *F. oxysporum.* Table S22. Gibberelin content in shoots of infected control flax plants and plants treated with Spd (10 mM and 100 mM) and infected with *F. oxysporum*. Table S23. Jasmonate, salicylic acid, and abscisic content in roots of infected control flax plants and plants treated with Spd (10 mM and 100 mM) and infected with *F. oxysporum*. Table S24. Jasmonate, salicylic acid, and abscisic content in shoots of infected control flax plants and plants treated with Spd (10 mM and 100 mM) and infected with *F. oxysporum.*

## Abbreviations

Cytokinins:	
*t*Z	*trans*-zeatin
*t*ZR	*trans*-zeatin riboside
*c*Z	*cis*-zeatin
*c*ZR	*cis*-zeatin riboside
DHZR	dihydrozeatin riboside
iP	N^6^—isopentenyladenine
iPR	N^6^—isopentenyladenosine
*t*ZROG	*trans*-zeatin riboside-*O*-glucoside
*c*ZOG	*cis*-zeatin-*O*-glucoside
cZROG	*cis*-zeatin riboside *O*-glucoside
DHZOG	dihydrozeatin-*O*-glucoside
DHZROG	dihydrozeatin riboside-*O*-glucoside
2MeScZR	2-methylthio-*cis*-zeatin riboside
Auxins:	
TRP	tryptophan
TRA	tryptamine
ANT	anthranilic acid
IAA	indole-3-acetic acid (active auxin)
oxIAA	2-oxindole-3-acetic acid (oxidised form of IAA)
IAAsp	indole-3-acetyl-aspartate (conjugated form of IAA)
Gibberellins:	
GA_1_, GA_3_, GA_4_	bioactive gibberellins
GA_53_, GA_44_, GA_19_, GA_20_	gibberellin biosynthetic precursors
GA_8_, GA_29_, GA_34_	gibberellin catabolites / deactivation products
Jasmonates:	
JA-Ile	jasmonoyl-isoleucine (bioactive form of JA)
JA	jasmonic acid
9,10-DHJA	9,10-dihydrojasmonic acid
12-OH-JA	12-hydroxy-jasmonic acid
*cis*-OPDA	12-oxo-phytodienoic acid (biosynthetic precursor of JA)
Salicylic acid:	
SA	salicylic acid
Abscisic acid:	
ABA	abscisic acid
