# Supplementary material for: Dynamic Hormonal Networks in Flax During Fusarium oxysporum Infection and Their Regulation by Spermidine
Source: Molecules. 2025 Dec 2;30(23):4631. doi: 10.3390/molecules30234631 (PMC12692866; doi:10.3390/molecules30234631)
Supplement: Supplementary file 1 [file molecules-30-04631-s001.zip › molecules-3990931-supplementary.pdf]

Supplementary tables

Table S1 Cytokinins content in roots of non-infected control flax plants and plants infected with *F. oxysporum*

**CKs [pmol/g FW]**	**2d**		**3d**		**7d**		**14d**	
	**Ctr**	**Foln**	**Ctr**	**Foln**	**Ctr**	**Foln**	**Ctr**	**Foln**
*t*ZR	0.23 ± 0.01	0.24 ± 0.06	0.18 ± 0.03	0.20 ± 0.01	0.44 ± 0.05	0.15 ± 0.01	0.25 ± 0.05	0.32 ± 0.02
*c*Z	1.02 ± 0.04	0.68 ± 0.05	0.75 ± 0.09	0.58 ± 0.09	0.39 ± 0.01	0.45 ± 0.01	0.52 ± 0.08	0.41 ± 0.02
*c*ZR	0.46 ± 0.03	0.40 ± 0.04	0.40 ± 0.04	0.33 ± 0.04	0.37 ± 0.04	0.42 ± 0.05	0.38 ± 0.01	0.89 ± 0.03
iP	1.00 ± 0.13	0.80 ± 0.02	0.64 ± 0.09	0.65 ± 0.18	0.55 ± 0.09	0.48 ± 0.01	0.78 ± 0.08	0.54 ± 0.02
iPR	0.22 ± 0.02	0.22 ± 0.03	0.20 ± 0.03	0.17 ± 0.02	0.24 ± 0.02	0.25 ± 0.02	0.51 ± 0.04	0.85 ± 0.06
*t*ZROG	0.15 ± 0.01	0.16 ± 0.01	0.16 ± 0.01	0.19 ± 0.01	0.23 ± 0.02	0.18 ± 0.01	0.15 ± 0.00	0.28 ± 0.00
*c*ZOG	13.49 ± 0.64	86.57 ± 6.91	11.60 ± 1.18	11.56 ± 0.13	7.15 ± 0.59	7.57 ± 0.39	9.26 ± 0.79	9.41 ± 0.31
*c*ZROG	4.94 ± 0.17	3.70 ± 0.60	3.81 ± 0.29	3.90 ± 0.43	3.98 ± 0.19	3.95 ± 0.17	3.60 ± 0.37	7.18 ± 0.31
DHZROG	nd.	nd.	nd.	nd.	nd.	nd.	0.15 ± 0.00	0.36 ± 0.01
2MeS*c*ZR	0.35 ± 0.02	0.34 ± 0.03	0.30 ± 0.00	0.26 ± 0.01	0.26 ± 0.02	0.23 ± 0.02	0.25 ± 0.01	0.41 ± 0.01

nd. not detected

Table S2 Cytokinins content in shoots of non-infected control flax plants and plants infected with *F. oxysporum*

**CKs [pmol/g FW]**	**2d**		**3d**		**7d**		**14d**	
	**Ctr**	**Foln**	**Ctr**	**Foln**	**Ctr**	**Foln**	**Ctr**	**Foln**
*t*Z	nd.	nd.	nd.	nd.	nd.	nd.	0.12 ± 0.01	0.14 ± 0.00
*t*ZR	0.07 ± 0.01	0.06 ± 0.02	0.05 ± 0.02	0.06 ± 0.02	0.08 ± 0.02	0.06 ± 0.01	0.14 ± 0.00	0.39 ± 0.01
*c*Z	0.09 ± 0.02	0.09 ± 0.02	0.10 ± 0.00	0.10 ± 0.00	0.09 ± 0.02	0.22 ± 0.01	0.11 ± 0.01	0.11 ± 0.01
*c*ZR	0.21 ± 0.00	0.26 ± 0.01	0.13 ± 0.01	0.17 ± 0.01	0.12 ± 0.01	0.15 ± 0.01	0.14 ± 0.01	0.19 ± 0.01
DHZR	0.01 ± 0.00	0.01 ± 0.00	0.03 ± 0.01	0.01 ± 0.01	nd.	nd.	0.03 ± 0.02	0.03 ± 0.01
iP	0.06 ± 0.00	0.09 ± 0.00	0.06 ± 0.01	0.07 ± 0.01	0.09 ± 0.01	0.07 ± 0.01	0.10 ± 0.02	0.16 ± 0.02
iPR	0.37 ± 0.04	0.27 ± 0.03	0.17 ± 0.04	0.21 ± 0.04	0.17 ± 0.03	0.19 ± 0.04	0.29 ± 0.05	0.29 ± 0.01
*t*ZROG	0.24 ± 0.00	0.26 ± 0.02	0.20 ± 0.01	0.27 ± 0.02	0.35 ± 0.02	0.31 ± 0.03	0.27 ± 0.01	0.39 ± 0.01
*c*ZOG	8.42 ± 1.39	6.50 ± 0.06	5.23 ± 0.66	6.89 ± 0.75	10.44 ± 0.36	7.72 ± 0.65	8.70 ± 1.03	10.07 ± 0.95
*c*ZROG	7.64 ± 0.63	7.49 ± 0.80	6.97 ± 0.49	9.39 ± 0.50	10.71 ± 0.14	8.68 ± 0.04	9.86 ± 0.44	12.54 ± 1.35
DHZOG	0.40 ± 0.04	0.54 ± 0.01	0.32 ± 0.02	0.54 ± 0.04	0.65 ± 0.03	0.51 ± 0.02	0.42 ± 0.04	0.56 ± 0.03
DHZROG	0.31 ± 0.02	0.34 ± 0.03	0.29 ± 0.02	0.37 ± 0.05	0.45 ± 0.03	0.36 ± 0.03	0.40 ± 0.03	0.50 ± 0.01
2MeS*c*ZR	1.02 ± 0.11	1.75 ± 0.01	1.09 ± 0.10	1.03 ± 0.04	1.13 ± 0.09	0.73 ± 0.07	0.69 ± 0.13	0.75 ± 0.03

nd. not detected

Table S3 Auxins and precursors content in roots of non-infected control flax plants and plants infected with *F. oxysporum*

AUX [pmol/g FW]	2d		3d		7d		14d	
	**Ctr**	**Foln**	**Ctr**	**Foln**	**Ctr**	**Foln**	**Ctr**	**Foln**
TRP	52158.09 ± 1402.00	36308.60 ± 7969.95	30053.14 ± 2858.98	29151.27 ± 126.35	19613.57 ± 1955.29	22636.33 ± 2071.39	17463.04 ± 1220.63	8849.38 ± 1520.78
TRA	1.89± 0.20	1.55 ± 0.10	1.11 ± 0.20	1.48 ± 0.04	1.93 ± 0.02	2.13 ± 0.12	2.63 ± 0.12	2.40 ± 0.44
ANT	0.72 ± 0.14	0.35 ± 0.09	0.58 ± 0.13	0.43 ± 0.14	4.30 ± 0.64	3.18 ± 0.41	4.35 ± 0.23	8.99 ± 0.58
IAA	9.92 ± 0.52	9.30 ± 0.62	9.28 ± 0.87	9.73 ± 0.30	22.39 ± 1.05	8.60 ± 0.36	10.37 ± 0.42	9.71 ± 0.29
oxIAA	5.29 ± 0.37	4.72 ± 0.36	5.63 ± 0.41	4.95 ± 0.23	6.56 ± 0.19	4.99 ± 0.83	2.68 ± 0.15	3.61 ± 0.13
IAAsp	0.70 ± 0.06	1.10 ± 0.12	1.06 ± 0.19	1.06 ± 0.20	0.73 ± 0.04	0.97 ± 0.13	0.82 ± 0.07	1.44 ± 0.03

Table S4 Auxins and precursors content in shoots of non-infected control flax plants and plants infected with *F. oxysporum*

AUX [pmol/g FW]	2d		3d		7d		14d	
	**Ctr**	**Foln**	**Ctr**	**Foln**	**Ctr**	**Foln**	**Ctr**	**Foln**
TRP	104167.31 ± 1696.27	195882.28 ± 4289.35	139565.43 ± 13107.57	116564.96 ± 11162.50	110521.45 ± 8958.98	101224.79 ± 17294.45	79254.80 ± 24322.91	52577.59 ± 6272.43
TRA	296.98 ± 64.20	171.76 ± 10.97	178.40 ± 25.13	284.44 ± 6.92	374.27 ± 45.18	410.24 ± 61.73	423.15 ± 7.14	433.81 ± 27.30
ANT	0.64 ± 0.27	3.61 ± 0.38	1.70 ± 0.18	0.98 ± 0.31	1.16 ± 0.06	3.41 ± 0.62	4.99 ± 0.69	4.29 ± 0.55
IAA	12.39 ± 1.63	11.08 ± 0.17	8.97 ± 0.33	11.81 ± 0.45	13.07 ± 1.20	9.92 ± 1.15	6.84 ± 0.43	7.24 ± 1.39
oxIAA	10.94 ± 0.19	11.48 ± 0.32	9.57 ± 0.87	9.23 ± 0.45	12.77 ± 0.32	7.70 ± 2.14	5.35 ± 0.65	3.91 ± 1.02
IAAsp	1.15 ± 0.10	1.26 ± 0.22	1.25 ± 0.10	2.55 ± 0.63	1.07 ± 0.15	2.25 ± 0.24	2.33 ± 0.19	2.66 ± 0.41

Table S5 Gibberellins content in roots of non-infected control flax plants and plants infected with *F. oxysporum*

GAs [pmol/g FW]	2d		3d		7d		14d	
	Ctr	Foln	Ctr	Foln	Ctr	Foln	Ctr	Foln
GA_4	0.03 ± 0.01	0.01 ± 0.01	0.02 ± 0.01	0.02 ± 0.00	0.02 ± 0.01	0.04 ± 0.01	0.01 ± 0.01	0.02 ± 0.01
GA_{34}	0.08 ± 0.01	0.05 ± 0.01	0.08 ± 0.01	0.08 ± 0.01	0.08 ± 0.01	0.08 ± 0.01	0.10 ± 0.02	0.07 ± 0.00
GA_{53}	0.01 ± 0.01	0.01 ± 0.00	0.01 ± 0.00	0.02 ± 0.00	0.02 ± 0.00	0.02 ± 0.00	0.03 ± 0.01	0.06 ± 0.01
GA_{44}	0.03 ± 0.01	0.04 ± 0.00	0.04 ± 0.00	0.04 ± 0.01	0.11 ± 0.00	0.15 ± 0.01	0.24 ± 0.03	3.89 ± 0.03
GA_{19}	0.12 ± 0.02	0.11 ± 0.01	0.13 ± 0.01	0.11 ± 0.02	0.15 ± 0.01	0.14 ± 0.01	0.14 ± 0.01	0.24 ± 0.01
GA_{20}	0.08 ± 0.01	0.17 ± 0.02	0.15 ± 0.01	0.16 ± 0.01	0.26 ± 0.00	0.21 ± 0.02	0.13 ± 0.00	1.81 ± 0.21
GA_1	0.04 ± 0.00	0.03 ± 0.00	0.03 ± 0.01	0.03 ± 0.01	0.05 ± 0.01	0.05 ± 0.00	0.02 ± 0.01	0.09 ± 0.01
GA_{29}	0.52 ± 0.03	0.17 ± 0.02	0.18 ± 0.01	0.25 ± 0.02	0.26 ± 0.02	0.29 ± 0.05	0.41 ± 0.05	0.43 ± 0.05
GA_8	2.46 ± 0.17	2.04 ± 0.03	1.84 ± 0.09	1.89 ± 0.24	2.87 ± 0.02	1.93 ± 0.06	2.28 ± 0.03	2.01 ± 0.14
GA_3	0.16 ± 0.01	0.11 ± 0.01	0.16 ± 0.01	0.16 ± 0.00	0.17 ± 0.00	0.17 ± 0.02	0.12 ± 0.01	0.12 ± 0.01

Table S6 Gibberellins content in shoots of non-infected control flax plants and plants infected with *F. oxysporum*

GAs [pmol/g FW]	2d		3d		7d		14d	
	**Ctr**	**Foln**	**Ctr**	**Foln**	**Ctr**	**Foln**	**Ctr**	**Foln**
GA_4	0.03 ± 0.01	0.03 ± 0.01	0.02 ± 0.00	0.02 ± 0.01	0.04 ± 0.01	0.03 ± 0.01	0.02 ± 0.00	0.02 ± 0.00
GA_{34}	0.01 ± 0.00	0.01 ± 0.00	0.01 ± 0.00	0.01 ± 0.00	0.02 ± 0.00	0.01 ± 0.00	0.01 ± 0.00	0.01 ± 0.00
GA_{53}	0.13 ± 0.05	0.29 ± 0.04	0.02 ± 0.00	0.01 ± 0.01	0.28 ± 0.04	0.33 ± 0.04	0.63 ± 0.10	0.58 ± 0.06
GA_{44}	0.04 ± 0.01	0.03 ± 0.01	0.04 ± 0.00	0.05 ± 0.01	0.06 ± 0.01	0.06 ± 0.01	0.08 ± 0.01	0.09 ± 0.01
GA_{19}	0.24 ± 0.02	0.29 ± 0.02	0.21 ± 0.02	0.28 ± 0.01	0.33 ± 0.02	0.33 ± 0.00	0.50 ± 0.04	0.42 ± 0.01
GA_{20}	0.15 ± 0.04	0.16 ± 0.01	0.17 ± 0.03	0.09 ± 0.01	0.12 ± 0.01	0.07 ± 0.01	0.08 ± 0.02	0.11 ± 0.01
GA_1	0.20 ± 0.01	0.16 ± 0.03	0.13 ± 0.01	0.18 ± 0.02	0.05 ± 0.02	0.06 ± 0.01	0.06 ± 0.03	0.06 ± 0.01
GA_{29}	0.03 ± 0.01	0.04 ± 0.01	0.03 ± 0.01	0.05 ± 0.01	0.04 ± 0.00	0.04 ± 0.01	0.06 ± 0.01	0.12 ± 0.01
GA_8	0.71 ± 0.00	0.91 ± 0.01	0.61 ± 0.01	0.65 ± 0.07	0.97 ± 0.05	0.95 ± 0.05	1.09 ± 0.02	1.20 ± 0.02
GA_3	0.23 ± 0.01	0.31 ± 0.02	0.11 ± 0.01	0.11 ± 0.01	0.25 ± 0.00	0.19 ± 0.01	0.18 ± 0.01	0.25 ± 0.01

Table S7 Jasmonates, salicylic acid and abscisic content in roots of non-infected control flax plants and plants infected with *F. oxysporum*

JAs + ABA [pmol/g FW]	2d		3d		7d		14d	
	Ctr	Foln	Ctr	Foln	Ctr	Foln	Ctr	Foln
JA	0.04 ± 0.01	0.07 ± 0.01	0.03 ± 0.01	0.04 ± 0.02	0.04 ± 0.01	0.04 ± 0.00	0.08 ± 0.01	0.19 ± 0.04
9.10-DHJA	0.32 ± 0.19	0.31 ± 0.17	0.09 ± 0.00	0.06 ± 0.02	0.07 ± 0.05	0.04 ± 0.02	nd.	0.14 ± 0.06
SA	9.55 ± 1.74	9.63 ± 1.44	4.49 ± 0.67	3.18 ± 0.13	13.86 ± 0.38	9.43 ± 0.35	5.69 ± 0.05	17.66 ± 1.62
cis-OPDA	653.78 ± 85.33	1814.15 ± 59.46	813.85 ± 158.01	1086.80 ± 84.40	787.58 ± 175.74	1963.90 ± 185.62	456.49 ± 78.42	1003.72 ± 162.85
ABA	0.32 ± 0.05	0.24 ± 0.00	0.34 ± 0.03	0.33 ± 0.04	0.12 ± 0.01	0.38 ± 0.05	0.47 ± 0.09	0.48 ± 0.09

nd. not detected

Table S8 Jasmonates, salicylic acid and abscisic content in shoots of non-infected control flax plants and plants infected with *F. oxysporum*

**JAs + ABA [pmol/g FW]**	**2d**		**3d**		**7d**		**14d**	
	**Ctr**	**Foln**	**Ctr**	**Foln**	**Ctr**	**Foln**	**Ctr**	**Foln**
JA-Ile	nd.	nd.	nd.	nd.	nd.	0.17 ± 0.00	0.12 ± 0.02	0.96 ± 0.02
JA	0.13 ± 0.01	0.09 ± 0.02	0.07 ± 0.01	0.18 ± 0.02	0.07 ± 0.02	0.05 ± 0.02	0.18 ± 0.00	0.09 ± 0.00
9.10-DHJA	0.18 ± 0.01	0.28 ± 0.01	0.07 ± 0.02	0.09 ± 0.02	0.04 ± 0.00	0.07 ± 0.02	nd.	nd.
12-OH-JA	2.92 ± 0.35	6.38 ± 1.68	2.44 ± 0.25	2.40 ± 0.01	3.46 ± 0.13	6.27 ± 0.68	5.50 ± 1.02	1.30 ± 0.00
SA	3.27 ± 0.59	2.36 ± 0.13	1.05 ± 0.23	1.07 ± 0.38	1.22 ± 0.11	2.26 ± 0.00	1.89 ± 0.15	3.04 ± 0.42
cis-OPDA	1898.71 ± 194.04	1873.39 ± 41.42	727.70 ± 70.77	1906.53 ± 420.23	1498.62 ± 94.10	1892.36 ± 67.52	1753.69 ± 171.14	2173.19 ± 69.60
ABA	0.91 ± 0.35	0.93 ± 0.11	1.05 ± 0.06	0.66 ± 0.13	1.77 ± 0.11	0.93 ± 0.11	1.42 ± 0.03	1.10 ± 0.02

nd. not detected

Table S9 Cytokinins content in roots of non-infected control flax plants and plants treated with Spd (10 mM and 100 mM)

CKs [pmol/g FW]	3d			4d			8d			15d		
	Ctr	10	100	Ctr	10	100	Ctr	10	100	Ctr	10	100
*t*ZR	0.23 ± 0.01	0.37 ± 0.01	0.26 ± 0.02	0.18 ± 0.03	0.31 ± 0.03	0.36 ± 0.02	0.44 ± 0.05	0.19 ± 0.01	0.51 ± 0.04	0.25 ± 0.05	0.40 ± 0.03	0.62 ± 0.00
*c*Z	1.02 ± 0.04	0.50 ± 0.02	0.45 ± 0.03	0.75 ± 0.09	0.40 ± 0.04	0.40 ± 0.10	0.39 ± 0.01	0.51 ± 0.04	0.36 ± 0.03	0.52 ± 0.08	0.34 ± 0.02	0.46 ± 0.06
*c*ZR	0.46 ± 0.03	0.41 ± 0.02	0.31 ± 0.04	0.40 ± 0.04	0.32 ± 0.02	0.39 ± 0.03	0.37 ± 0.04	0.26 ± 0.04	0.37 ± 0.03	0.38 ± 0.01	0.31 ± 0.00	0.27 ± 0.02
iP	1.00 ± 0.13	0.96 ± 0.05	0.88 ± 0.07	0.64 ± 0.09	0.46 ± 0.08	1.75 ± 0.12	0.55 ± 0.09	0.60 ± 0.09	0.66 ± 0.07	0.78 ± 0.08	0.51 ± 0.03	0.55 ± 0.03
iPR	0.22 ± 0.02	0.26 ± 0.01	0.33 ± 0.01	0.20 ± 0.03	0.21 ± 0.03	0.23 ± 0.03	0.24 ± 0.02	0.16 ± 0.03	0.35 ± 0.01	0.51 ± 0.04	0.48 ± 0.03	0.51 ± 0.05
*t*ZROG	0.15 ± 0.01	0.21 ± 0.00	0.16 ± 0.02	0.16 ± 0.01	0.13 ± 0.02	0.25 ± 0.03	0.23 ± 0.02	0.10 ± 0.03	0.24 ± 0.02	0.15 ± 0.00	0.19 ± 0.01	0.23 ± 0.02
*c*ZOG	13.49 ± 0.64	8.55 ± 0.35	2.49 ± 0.05	11.60 ± 1.18	11.30 ± 0.46	8.67 ± 0.41	7.15 ± 0.59	8.13 ± 0.64	7.56 ± 0.54	9.26 ± 0.79	7.07 ± 0.52	6.22 ± 0.38
*c*ZROG	4.94 ± 0.17	3.90 ± 0.26	3.82 ± 0.17	3.81 ± 0.29	4.98 ± 0.57	4.11 ± 0.35	3.98 ± 0.19	2.89 ± 0.28	3.61 ± 0.18	3.60 ± 0.37	4.43 ± 0.51	3.88 ± 0.51
DHZROG	nd.	nd.	nd.	nd.	nd.	nd.	nd.	nd.	nd.	0.15 ± 0.00	0.15 ± 0.00	0.15 ± 0.00
2MeScZR	0.35 ± 0.02	0.24 ± 0.02	0.22 ± 0.04	0.30 ± 0.00	0.23 ± 0.02	0.21 ± 0.02	0.26 ± 0.02	0.21 ± 0.02	0.21 ± 0.03	0.25 ± 0.01	0.24 ± 0.02	0.23 ± 0.02

nd. not detected

Table S10 Cytokinins content in shoots of non-infected control flax plants and plants treated with Spd (10 mM and 100 mM)

CKs [pmol/g FW]	3d			4d			8d			15d		
	**Ctr**	**10**	**100**	**Ctr**	**10**	**100**	**Ctr**	**10**	**100**	**Ctr**	**10**	**100**
*t*Z	nd.	nd.	nd.	nd.	nd.	nd.	nd.	nd.	nd.	0.12 ± 0.01	0.17 ± 0.01	0.14 ± 0.01
*t*ZR	0.07 ± 0.01	0.09 ± 0.02	0.09 ± 0.01	0.05 ± 0.02	0.09 ± 0.01	0.07 ± 0.01	0.08 ± 0.02	0.07 ± 0.02	0.15 ± 0.01	0.14 ± 0.00	0.56 ± 0.02	0.39 ± 0.05
*c*Z	0.09 ± 0.02	0.10 ± 0.02	0.08 ± 0.01	0.10 ± 0.00	0.13 ± 0.00	0.08 ± 0.01	0.09 ± 0.02	0.13 ± 0.00	0.12 ± 0.00	0.11 ± 0.01	0.09 ± 0.01	0.09 ± 0.00
*c*ZR	0.21 ± 0.00	0.17 ± 0.02	0.17 ± 0.02	0.13 ± 0.01	0.17 ± 0.02	0.20 ± 0.03	0.12 ± 0.01	0.14 ± 0.01	0.16 ± 0.01	0.14 ± 0.01	0.14 ± 0.01	0.13 ± 0.01
DHZR	0.01 ± 0.00	0.03 ± 0.02	0.03 ± 0.02	0.03 ± 0.01	0.02 ± 0.01	0.02 ± 0.00	nd.	nd.	nd.	0.03 ± 0.02	0.02 ± 0.00	0.03 ± 0.00
iP	0.06 ± 0.00	0.09 ± 0.01	0.08 ± 0.01	0.06 ± 0.01	0.06 ± 0.01	0.04 ± 0.01	0.09 ± 0.01	0.07 ± 0.00	0.10 ± 0.01	0.10 ± 0.02	0.12 ± 0.01	0.19 ± 0.02
iPR	0.37 ± 0.04	0.36 ± 0.02	0.28 ± 0.04	0.17 ± 0.04	0.23 ± 0.02	0.26 ± 0.01	0.17 ± 0.03	0.19 ± 0.02	0.35 ± 0.03	0.29 ± 0.05	0.54 ± 0.03	0.38 ± 0.00
*t*ZROG	0.24 ± 0.00	0.33 ± 0.01	0.26 ± 0.02	0.20 ± 0.01	0.27 ± 0.01	0.27 ± 0.01	0.35 ± 0.02	0.30 ± 0.01	0.33 ± 0.01	0.27 ± 0.01	0.40 ± 0.05	0.40 ± 0.04
*c*ZOG	8.42 ± 1.39	9.42 ± 1.46	6.07 ± 1.68	5.23 ± 0.66	6.71 ± 0.27	4.80 ± 0.78	10.44 ± 0.36	8.89 ± 0.81	8.56 ± 0.63	8.70 ± 1.03	10.31 ± 0.56	12.76 ± 0.44
*c*ZROG	7.64 ± 0.63	8.56 ± 0.81	7.78 ± 0.24	6.97 ± 0.49	9.23 ± 0.79	7.52 ± 0.65	10.71 ± 0.14	8.49 ± 0.14	9.13 ± 0.06	9.86 ± 0.44	11.35 ± 1.02	11.39 ± 0.51
DHZOG	0.40 ± 0.04	0.56 ± 0.01	0.56 ± 0.03	0.32 ± 0.02	0.54 ± 0.03	0.60 ± 0.00	0.65 ± 0.03	0.49 ± 0.01	0.61 ± 0.02	0.42 ± 0.04	0.62 ± 0.01	0.54 ± 0.04
DHZROG	0.31 ± 0.02	0.35 ± 0.00	0.33 ± 0.01	0.29 ± 0.02	0.31 ± 0.03	0.31 ± 0.03	0.45 ± 0.03	0.38 ± 0.02	0.39 ± 0.01	0.40 ± 0.03	0.50 ± 0.03	0.49 ± 0.01
2MeScZR	1.02 ± 0.11	1.05 ± 0.02	1.07 ± 0.01	1.09 ± 0.10	1.16 ± 0.03	1.67 ± 0.11	1.13 ± 0.09	1.33 ± 0.09	0.83 ± 0.03	0.69 ± 0.13	0.79 ± 0.01	0.51 ± 0.01

nd. not detected

Table S11 Auxins and precursors content in roots of non-infected control flax plants and plants treated with Spd (10 mM and 100 mM)

AUX [pmol/g FW]	3d			4d			8d			15d		
	Ctr	10	100	Ctr	10	100	Ctr	10	100	Ctr	10	100
TRP	52158.09 ± 1402.00	14864.14 ± 338.85	18999.84 ± 263.49	30053.14 ± 2858.98	18368.26 ± 2092.59	16404.63 ± 3694.71	19613.57 ± 1955.29	22152.46 ± 1204.58	20367.63 ± 2913.22	17463.04 ± 1220.63	16828.63 ± 671.53	18560.60 ± 2260.26
TRA	1.89± 0.20	6.68 ± 0.15	1.24 ± 0.10	1.11 ± 0.20	1.25 ± 0.21	1.38 ± 0.09	1.93 ± 0.02	1.55 ± 0.01	1.76 ± 0.08	2.63 ± 0.12	3.92 ± 0.61	1.76 ± 0.20
ANT	0.72 ± 0.14	3.16 ± 0.20	0.31 ± 0.02	0.58 ± 0.13	0.36 ± 0.02	1.18 ± 0.00	4.30 ± 0.64	1.33 ± 0.91	2.57 ± 0.41	4.35 ± 0.23	7.04 ± 0.71	9.37 ± 0.91
IAA	9.92 ± 0.52	11.36 ± 0.31	10.48 ± 0.77	9.28 ± 0.87	11.52 ± 0.89	10.28 ± 0.62	22.39 ± 1.05	8.28 ± 0.28	12.44 ± 0.43	10.37 ± 0.42	9.03 ± 0.52	8.26 ± 0.16
oxIAA	5.29 ± 0.37	4.42 ± 0.27	3.90 ± 0.25	5.63 ± 0.41	4.22 ± 0.67	5.55 ± 0.47	6.56 ± 0.19	3.97 ± 0.23	4.50 ± 0.24	2.68 ± 0.15	2.83 ± 0.05	2.53 ± 0.13
IAAsp	0.70 ± 0.06	0.76 ± 0.06	1.00 ± 0.06	1.06 ± 0.19	1.19 ± 0.24	1.05 ± 0.11	0.73 ± 0.04	0.84 ± 0.03	0.73 ± 0.13	0.82 ± 0.07	0.79 ± 0.16	3.31 ± 0.13

Table S12 Auxins and precursors content in shoots of non-infected control flax plants and plants treated with Spd (10 mM and 100 mM)

AUX [pmol/g FW]	3d			4d			8d			15d		
	**Ctr**	**10**	**100**	**Ctr**	**10**	**100**	**Ctr**	**10**	**100**	**Ctr**	**10**	**100**
TRP	104167.31 ± 1696.27	116010.02 ± 14845.09	96694.99 ± 7872.63	139565.43 ± 13107.57	138636.37 ± 4768.24	143742.88 ± 3522.25	110521.45 ± 8958.98	129036.89 ± 14070.01	83801.74 ± 1626.99	79254.80 ± 24322.91	93279.95 ± 10824.98	69409.59 ± 11227.20
TRA	296.98 ± 64.20	269.52 ± 56.58	317.22 ± 42.70	178.40 ± 25.13	316.03 ± 1.62	288.69 ± 22.27	374.27 ± 45.18	489.54 ± 46.64	394.74 ± 23.66	423.15 ± 7.14	512.00 ± 44.10	379.51 ± 41.06
ANT	0.64 ± 0.27	1.34 ± 0.66	2.33 ± 0.83	1.70 ± 0.18	3.72 ± 0.23	1.50 ± 0.44	1.16 ± 0.06	1.33 ± 0.68	1.83 ± 0.12	4.99 ± 0.69	3.61 ± 0.28	1.73 ± 0.17
IAA	12.39 ± 1.63	15.13 ± 1.64	8.08 ± 0.87	8.97 ± 0.33	11.80 ± 0.31	10.65 ± 0.80	13.07 ± 1.20	13.04 ± 1.07	10.13 ± 0.85	6.84 ± 0.43	9.46 ± 0.41	9.02 ± 1.06
oxIAA	10.94 ± 0.19	13.59 ± 0.37	5.98 ± 0.80	9.57 ± 0.87	8.91 ± 1.15	12.55 ± 0.68	12.77 ± 0.32	13.60 ± 1.56	6.57 ± 0.73	5.35 ± 0.65	5.53 ± 0.69	3.87 ± 0.59
IAAsp	1.15 ± 0.10	1.23 ± 0.31	1.84 ± 0.35	1.25 ± 0.10	1.03 ± 0.12	0.98 ± 0.21	1.07 ± 0.15	1.63 ± 0.11	1.13 ± 0.05	2.33 ± 0.19	2.85 ± 0.41	3.22 ± 0.74

Table S13 Gibberellins content in roots of non-infected control flax plants and plants treated with Spd (10 mM and 100 mM)

GAs [pmol/g FW]	3d			4d			8d			15d		
	Ctr	10	100	Ctr	10	100	Ctr	10	100	Ctr	10	100
GA_4	0.03 ± 0.01	0.03 ± 0.01	0.02 ± 0.00	0.02 ± 0.01	0.03 ± 0.01	0.02 ± 0.01	0.02 ± 0.01	0.02 ± 0.00	0.06 ± 0.01	0.01 ± 0.01	0.02 ± 0.00	0.01 ± 0.00
GA_{34}	0.08 ± 0.01	0.05 ± 0.00	0.04 ± 0.01	0.08 ± 0.01	0.06 ± 0.01	0.05 ± 0.01	0.08 ± 0.01	0.09 ± 0.01	0.05 ± 0.01	0.10 ± 0.02	0.05 ± 0.00	0.04 ± 0.00
GA_{53}	0.01 ± 0.01	0.02 ± 0.00	0.01 ± 0.00	0.01 ± 0.00	0.02 ± 0.00	0.02 ± 0.01	0.02 ± 0.00	0.01 ± 0.00	0.03 ± 0.01	0.03 ± 0.01	0.08 ± 0.01	0.08 ± 0.00
GA_{44}	0.03 ± 0.01	0.04 ± 0.00	0.04 ± 0.01	0.04 ± 0.00	0.08 ± 0.00	0.03 ± 0.01	0.11 ± 0.00	0.06 ± 0.01	0.90 ± 0.13	0.24 ± 0.03	1.37 ± 0.08	0.42 ± 0.11
GA_{19}	0.12 ± 0.02	0.09 ± 0.01	0.10 ± 0.01	0.13 ± 0.01	0.14 ± 0.01	0.11 ± 0.01	0.15 ± 0.01	0.09 ± 0.00	0.14 ± 0.02	0.14 ± 0.01	0.23 ± 0.02	0.20 ± 0.02
GA_{20}	0.08 ± 0.01	0.16 ± 0.02	0.12 ± 0.01	0.15 ± 0.01	0.20 ± 0.01	0.22 ± 0.01	0.26 ± 0.00	0.26 ± 0.00	0.09 ± 0.00	0.13 ± 0.00	0.44 ± 0.03	0.23 ± 0.01
GA_1	0.04 ± 0.00	0.01 ± 0.01	0.02 ± 0.00	0.03 ± 0.01	0.03 ± 0.01	0.03 ± 0.01	0.05 ± 0.01	0.03 ± 0.01	0.04 ± 0.01	0.02 ± 0.01	0.08 ± 0.01	0.05 ± 0.01
GA_{29}	0.52 ± 0.03	0.12 ± 0.02	0.17 ± 0.02	0.18 ± 0.01	0.15 ± 0.01	0.13 ± 0.01	0.26 ± 0.02	0.25 ± 0.03	0.31 ± 0.02	0.41 ± 0.05	0.35 ± 0.02	0.18 ± 0.03
GA_8	2.46 ± 0.17	2.90 ± 0.04	1.59 ± 0.14	1.84 ± 0.09	2.18 ± 0.22	2.61 ± 0.19	2.87 ± 0.02	2.09 ± 0.05	2.03 ± 0.14	2.28 ± 0.03	1.87 ± 0.15	1.56 ± 0.16
GA_3	0.16 ± 0.01	0.15 ± 0.01	0.08 ± 0.01	0.16 ± 0.01	0.17 ± 0.01	0.14 ± 0.01	0.17 ± 0.00	0.16 ± 0.00	0.16 ± 0.01	0.12 ± 0.01	0.11 ± 0.01	0.14 ± 0.01

Table S14 Gibberellins content in shoots of non-infected control flax plants and plants treated with Spd (10 mM and 100 mM)

GAs [pmol/g FW]	3d			4d			8d			15d		
	Ctr	10	100	Ctr	10	100	Ctr	10	100	Ctr	10	100
GA_4	0.03 ± 0.01	0.02 ± 0.01	0.02 ± 0.00	0.02 ± 0.00	0.02 ± 0.01	0.02 ± 0.01	0.04 ± 0.01	0.03 ± 0.01	0.05 ± 0.02	0.02 ± 0.00	0.03 ± 0.00	0.02 ± 0.01
GA_{34}	0.01 ± 0.00	0.01 ± 0.00	0.01 ± 0.00	0.01 ± 0.00	0.01 ± 0.00	0.02 ± 0.00	0.02 ± 0.00	0.01 ± 0.00	0.01 ± 0.00	0.01 ± 0.00	0.01 ± 0.00	0.01 ± 0.00
GA_{53}	0.13 ± 0.05	0.17 ± 0.05	0.16 ± 0.03	0.02 ± 0.00	0.02 ± 0.01	0.02 ± 0.01	0.28 ± 0.04	0.37 ± 0.02	0.36 ± 0.02	0.63 ± 0.10	0.44 ± 0.05	0.54 ± 0.09
GA_{44}	0.04 ± 0.01	0.03 ± 0.01	0.05 ± 0.02	0.04 ± 0.00	0.04 ± 0.01	0.05 ± 0.01	0.06 ± 0.01	0.03 ± 0.02	0.08 ± 0.02	0.08 ± 0.01	0.06 ± 0.01	0.08 ± 0.02
GA_{19}	0.24 ± 0.02	0.32 ± 0.02	0.21 ± 0.01	0.21 ± 0.02	0.25 ± 0.01	0.27 ± 0.01	0.33 ± 0.02	0.38 ± 0.01	0.31 ± 0.01	0.50 ± 0.04	0.44 ± 0.03	0.45 ± 0.03
GA_{20}	0.15 ± 0.04	0.08 ± 0.01	0.12 ± 0.01	0.17 ± 0.03	0.08 ± 0.03	0.08 ± 0.02	0.12 ± 0.01	0.14 ± 0.01	0.08 ± 0.02	0.08 ± 0.02	0.07 ± 0.01	0.07 ± 0.01
GA_1	0.20 ± 0.01	0.32 ± 0.02	0.16 ± 0.01	0.13 ± 0.01	0.15 ± 0.03	0.17 ± 0.01	0.05 ± 0.02	0.09 ± 0.03	0.05 ± 0.01	0.06 ± 0.03	0.03 ± 0.01	0.04 ± 0.01
GA_{29}	0.03 ± 0.01	0.03 ± 0.01	0.04 ± 0.01	0.03 ± 0.01	0.03 ± 0.01	0.03 ± 0.01	0.04 ± 0.00	0.11 ± 0.01	0.04 ± 0.01	0.06 ± 0.01	0.10 ± 0.02	0.06 ± 0.01
GA_8	0.71 ± 0.00	0.84 ± 0.04	0.63 ± 0.02	0.61 ± 0.01	0.98 ± 0.06	0.71 ± 0.05	0.97 ± 0.05	1.01 ± 0.04	0.76 ± 0.04	1.09 ± 0.02	1.02 ± 0.00	0.75 ± 0.03
GA_3	0.23 ± 0.01	0.19 ± 0.01	0.33 ± 0.01	0.11 ± 0.01	0.13 ± 0.01	0.12 ± 0.02	0.25 ± 0.00	0.19 ± 0.00	0.17 ± 0.01	0.18 ± 0.01	0.23 ± 0.02	0.29 ± 0.00

Table S15 Jasmonates. salicylic acid. and abscisic content in roots of non-infected control flax plants and plants treated with Spd (10 mM and 100 mM)

**JAs + ABA [pmol/g FW]**	**3d**			**4d**			**8d**			**15d**		
	**Ctr**	**10**	**100**	**Ctr**	**10**	**100**	**Ctr**	**10**	**100**	**Ctr**	**10**	**100**
JA	0.04 ± 0.01	0.03 ± 0.01	0.13 ± 0.01	0.03 ± 0.01	0.05 ± 0.01	0.04 ± 0.00	0.04 ± 0.01	0.03 ± 0.01	0.03 ± 0.00	0.08 ± 0.01	0.06 ± 0.01	0.04 ± 0.01
9.10-DHJA	0.32 ± 0.19	0.19 ± 0.15	0.57 ± 0.11	0.09 ± 0.00	0.08 ± 0.00	0.22 ± 0.01	0.07 ± 0.05	0.06 ± 0.05	0.05 ± 0.03	nd.	nd.	nd.
SA	9.55 ± 1.74	10.29 ± 2.60	4.59 ± 1.72	4.49 ± 0.67	10.78 ± 2.17	6.74 ± 0.35	13.86 ± 0.38	10.80 ± 3.07	18.95 ± 0.85	5.69 ± 0.05	20.59 ± 0.87	14.22 ± 0.23
*cis*OPDA	653.78 ± 85.33	1622.32 ± 25.64	1538.73 ± 265.67	813.85 ± 158.01	1002.57 ± 267.54	1985.30 ± 224.44	787.58 ± 175.74	597.59 ± 99.31	479.89 ± 67.52	456.49 ± 78.42	1471.65 ± 140.20	1172.79 ± 163.24
ABA	0.32 ± 0.05	0.43 ± 0.04	0.41 ± 0.04	0.34 ± 0.03	0.39 ± 0.03	0.50 ± 0.04	0.12 ± 0.01	0.31 ± 0.02	0.55 ± 0.03	0.47 ± 0.09	0.28 ± 0.04	0.55 ± 0.01

nd. not detected

Table S16 Jasmonates, salicylic acid and abscisic content in shoots of non-infected control flax plants and plants treated with Spd (10 mM and 100 mM)

JAs + ABA [pmol/g FW]	3d			4d			8d			15d		
	Ctr	10	100	Ctr	10	100	Ctr	10	100	Ctr	10	100
JA-Ile	nd.	nd.	nd.	nd.	nd.	nd.	nd.	nd.	nd.	0.12 ± 0.02	0.10 ± 0.00	0.17 ± 0.04
JA	0.13 ± 0.01	0.09 ± 0.01	0.26 ± 0.03	0.07 ± 0.01	0.12 ± 0.01	0.14 ± 0.04	0.07 ± 0.02	0.06 ± 0.00	0.09 ± 0.02	0.18 ± 0.00	0.09 ± 0.01	0.02 ± 0.00
9.10-DHJA	0.18 ± 0.01	0.30 ± 0.01	0.03 ± 0.01	0.07 ± 0.02	0.09 ± 0.00	0.14 ± 0.02	0.04 ± 0.00	0.07 ± 0.01	0.08 ± 0.03	nd.	nd.	nd.
12-OH-JA	2.92 ± 0.35	1.86 ± 0.79	1.30 ± 0.00	2.44 ± 0.25	3.08 ± 0.50	2.41 ± 1.57	3.46 ± 0.13	20.70 ± 0.01	1.30 ± 0.00	5.50 ± 1.02	1.30 ± 0.00	1.30 ± 0.00
SA	3.27 ± 0.59	4.35 ± 0.71	5.83 ± 0.51	1.05 ± 0.23	1.19 ± 0.11	3.90 ± 0.33	1.22 ± 0.11	2.69 ± 0.62	1.68 ± 0.50	1.89 ± 0.15	0.59 ± 0.57	2.25 ± 0.16
*cis*OPDA	1898.71 ± 194.04	1529.41 ± 229.16	3022.37 ± 163.94	727.70 ± 70.77	1097.39 ± 28.07	2412.01 ± 109.59	1498.62 ± 94.10	2708.47 ± 116.19	1629.88 ± 253.36	1753.69 ± 171.14	1709.73 ± 61.00	1304.32 ± 137.00
ABA	0.91 ± 0.35	0.98 ± 0.22	0.92 ± 0.13	1.05 ± 0.06	1.25 ± 0.03	1.07 ± 0.09	1.77 ± 0.11	1.02 ± 0.18	1.13 ± 0.02	1.42 ± 0.03	1.18 ± 0.08	1.12 ± 0.10

nd. not detected

Table S17 Cytokinins content in roots of infected control flax plants and plants treated with Spd (10 mM and 100 mM) and infected with *F. oxysporum*

CKs [pmol/g FW]	2d			3d			7d			14d		
	**Foln**	**10**	**100**	**Foln**	**10**	**100**	**Foln**	**10**	**100**	**Foln**	**10**	**100**
*t*ZR	0.24 ± 0.06	0.24 ± 0.01	0.29 ± 0.03	0.20 ± 0.01	0.20 ± 0.01	0.25 ± 0.01	0.15 ± 0.01	0.20 ± 0.02	0.32 ± 0.04	0.32 ± 0.02	0.42 ± 0.01	0.47 ± 0.02
*c*Z	0.68 ± 0.05	0.42 ± 0.03	0.58 ± 0.04	0.58 ± 0.09	0.58 ± 0.05	0.32 ± 0.02	0.45 ± 0.01	0.43 ± 0.04	0.25 ± 0.01	0.41 ± 0.02	0.36 ± 0.06	0.34 ± 0.04
*c*ZR	0.40 ± 0.04	0.32 ± 0.02	0.39 ± 0.00	0.33 ± 0.04	0.32 ± 0.05	0.26 ± 0.02	0.42 ± 0.05	0.38 ± 0.04	0.41 ± 0.02	0.89 ± 0.03	0.55 ± 0.00	0.63 ± 0.04
iP	0.80 ± 0.02	0.75 ± 0.09	0.65 ± 0.06	0.65 ± 0.18	0.59 ± 0.09	0.48 ± 0.08	0.48 ± 0.01	0.41 ± 0.01	0.38 ± 0.03	0.54 ± 0.02	0.66 ± 0.06	0.45 ± 0.03
iPR	0.22 ± 0.03	0.23 ± 0.04	0.22 ± 0.01	0.17 ± 0.02	0.17 ± 0.05	0.21 ± 0.03	0.25 ± 0.02	0.30 ± 0.02	0.34 ± 0.02	0.85 ± 0.06	0.67 ± 0.05	0.88 ± 0.02
*t*ZROG	0.16 ± 0.01	0.17 ± 0.01	0.23 ± 0.02	0.19 ± 0.01	0.16 ± 0.02	0.22 ± 0.02	0.18 ± 0.01	0.19 ± 0.01	0.22 ± 0.02	0.28 ± 0.00	0.32 ± 0.01	0.36 ± 0.01
*c*ZOG	86.57 ± 6.91	68.61 ± 3.88	9.92 ± 0.42	11.56 ± 0.13	10.33 ± 1.16	8.38 ± 0.66	7.57 ± 0.39	6.56 ± 0.03	7.09 ± 0.45	9.41 ± 0.31	6.87 ± 0.99	7.71 ± 1.12
*c*ZROG	3.70 ± 0.60	4.04 ± 0.30	3.38 ± 0.42	3.90 ± 0.43	3.22 ± 0.14	3.91 ± 0.19	3.95 ± 0.17	3.98 ± 0.41	4.04 ± 0.10	7.18 ± 0.31	6.86 ± 0.13	8.92 ± 0.57
DHZROG	nd.	nd.	nd.	nd.	nd.	nd.	nd.	nd.	nd.	0.36 ± 0.01	0.42 ± 0.03	0.56 ± 0.02
2MeScZR	0.34 ± 0.03	0.20 ± 0.01	0.25 ± 0.01	0.26 ± 0.01	0.27 ± 0.01	0.20 ± 0.02	0.23 ± 0.02	0.21 ± 0.00	0.23 ± 0.01	0.41 ± 0.01	0.32 ± 0.00	0.48 ± 0.05

nd. not detected

Table S18 Cytokinins content in shoots of infected control flax plants and plants treated with Spd (10 mM and 100 mM) and infected with *F. oxysporum*

CKs [pmol/g FW]	2d			3d			7d			14d		
	**Foln**	**10**	**100**	**Foln**	**10**	**100**	**Foln**	**10**	**100**	**Foln**	**10**	**100**
*t*Z	nd.	nd.	nd.	nd.	nd.	nd.	nd.	nd.	nd.	0.14 ± 0.00	0.16 ± 0.01	0.18 ± 0.01
*t*ZR	0.06 ± 0.02	0.11 ± 0.02	0.13 ± 0.02	0.06 ± 0.02	0.08 ± 0.02	0.08 ± 0.02	0.06 ± 0.01	0.06 ± 0.01	0.13 ± 0.00	0.39 ± 0.01	0.35 ± 0.02	0.54 ± 0.17
*c*Z	0.09 ± 0.02	0.13 ± 0.02	0.09 ± 0.03	0.10 ± 0.00	0.13 ± 0.01	0.08 ± 0.00	0.22 ± 0.01	0.10 ± 0.01	0.10 ± 0.02	0.11 ± 0.01	0.12 ± 0.01	0.11 ± 0.02
*c*ZR	0.26 ± 0.01	0.23 ± 0.01	0.24 ± 0.03	0.17 ± 0.01	0.19 ± 0.02	0.19 ± 0.00	0.15 ± 0.01	0.13 ± 0.02	0.16 ± 0.01	0.19 ± 0.01	0.19 ± 0.01	0.19 ± 0.02
DHZR	0.01 ± 0.00	0.03 ± 0.02	0.02 ± 0.00	0.01 ± 0.01	0.05 ± 0.00	0.11 ± 0.01	nd.	nd.	nd.	0.03 ± 0.01	0.03 ± 0.01	0.02 ± 0.00
iP	0.09 ± 0.00	0.34 ± 0.01	0.07 ± 0.01	0.07 ± 0.01	0.09 ± 0.00	0.06 ± 0.01	0.07 ± 0.01	0.08 ± 0.01	0.14 ± 0.02	0.16 ± 0.02	0.14 ± 0.01	0.16 ± 0.01
*i*PR	0.27 ± 0.03	0.32 ± 0.01	0.33 ± 0.02	0.21 ± 0.04	0.26 ± 0.03	0.21 ± 0.02	0.19 ± 0.04	0.14 ± 0.01	0.21 ± 0.03	0.29 ± 0.01	0.61 ± 0.06	0.59 ± 0.04
*t*ZROG	0.26 ± 0.02	0.32 ± 0.02	0.32 ± 0.03	0.27 ± 0.02	0.25 ± 0.03	0.32 ± 0.02	0.31 ± 0.03	0.26 ± 0.03	0.35 ± 0.03	0.39 ± 0.01	0.35 ± 0.03	0.43 ± 0.04
*c*ZOG	6.50 ± 0.06	9.70 ± 0.35	7.39 ± 0.39	6.89 ± 0.75	6.68 ± 0.29	7.70 ± 0.81	7.72 ± 0.65	7.01 ± 0.29	11.44 ± 0.49	10.07 ± 0.95	10.22 ± 0.84	11.48 ± 0.34
*c*ZROG	7.49 ± 0.80	8.77 ± 0.67	7.56 ± 0.69	9.39 ± 0.50	7.96 ± 0.68	8.84 ± 0.40	8.68 ± 0.04	8.28 ± 0.58	12.41 ± 0.14	12.54 ± 1.35	10.06 ± 1.15	14.23 ± 1.19
DHZOG	0.54 ± 0.01	0.53 ± 0.04	0.54 ± 0.01	0.54 ± 0.04	0.59 ± 0.03	0.57 ± 0.05	0.51 ± 0.02	0.42 ± 0.04	0.34 ± 0.01	0.56 ± 0.03	0.62 ± 0.04	0.65 ± 0.02
DHZROG	0.34 ± 0.03	0.41 ± 0.02	0.37 ± 0.03	0.37 ± 0.05	0.35 ± 0.04	0.36 ± 0.03	0.36 ± 0.03	0.36 ± 0.04	0.57 ± 0.00	0.50 ± 0.01	0.44 ± 0.02	0.62 ± 0.02
2MeScZR	1.75 ± 0.01	1.29 ± 0.13	1.23 ± 0.01	1.03 ± 0.04	1.20 ± 0.03	1.44 ± 0.10	0.73 ± 0.07	0.80 ± 0.04	0.78 ± 0.14	0.75 ± 0.03	0.71 ± 0.03	0.90 ± 0.07

nd. not detected

Table S19 Auxins and precursors content in roots of infected control flax plants and plants treated with Spd (10 mM and 100 mM) and infected with *F. oxysporum*

AUX [pmol/g FW]	2d			3d			7d			14d		
	**Foln**	**10**	**100**	**Foln**	**10**	**100**	**Foln**	**10**	**100**	**Foln**	**10**	**100**
TRP	36308.60 ± 7969.95	9764.45 ± 199.02	31010.24 ± 3722.80	29151.27 ± 126.35	15056.60 ± 3523.75	23465.62 ± 762.82	22636.33 ± 2071.39	31736.34 ± 610.28	37123.73 ± 1568.84	8849.38 ± 1520.78	6785.63 ± 1043.36	10831.67 ± 1818.20
TRA	1.55 ± 0.10	1.16 ± 0.08	1.17 ± 0.02	1.48 ± 0.04	3.88 ± 0.40	1.33 ± 0.01	2.13 ± 0.12	2.30 ± 0.15	1.71 ± 0.28	2.40 ± 0.44	2.89 ± 0.19	1.92 ± 0.24
ANT	0.35 ± 0.09	0.33 ± 0.03	0.19 ± 0.02	0.43 ± 0.14	1.11 ± 0.30	0.60 ± 0.02	3.18 ± 0.41	2.29 ± 0.04	3.11± 0.15	8.99 ± 0.58	12.56 ± 1.09	8.59 ± 1.28
IAA	9.30 ± 0.62	9.90 ± 0.45	10.06 ± 0.33	9.73 ± 0.30	10.95 ± 0.66	9.06 ± 0.10	8.60 ± 0.36	7.64 ± 0.40	10.73 ± 0.39	9.71 ± 0.29	8.15 ± 0.32	9.50 ± 0.24
oxIAA	4.72 ± 0.36	3.68 ± 0.34	3.37 ± 0.30	4.95 ± 0.23	4.55 ± 0.13	4.16 ± 0.30	4.99 ± 0.83	3.28 ± 0.24	6.18 ± 0.57	3.61 ± 0.13	2.06 ± 0.03	3.11 ± 0.28
IAAsp	1.10 ± 0.12	0.60 ± 0.09	0.56 ± 0.20	1.06 ± 0.20	0.60 ± 0.18	0.90 ± 0.13	0.97 ± 0.13	0.95 ± 0.11	1.36 ± 0.14	1.44 ± 0.03	0.67 ± 0.09	1.54 ± 0.09

Table S20 Auxins and precursors content in shoots of infected control flax plants and plants treated with Spd (10 mM and 100 mM) and infected with *F. oxysporum*

**AUX [pmol/g FW]**	**2d**			**3d**			**7d**			**14d**		
	**Foln**	**10**	**100**	**Foln**	**10**	**100**	**Foln**	**10**	**100**	**Foln**	**10**	**100**
TRP	195882.28 ± 4289.35	145312.24 ± 17442.73	109725.64 ± 8135.20	116564.96 ± 11162.50	157497.21 ± 10104.93	145907.05 ± 9442.15	101224.79 ± 17294.45	102356.58 ± 10611.28	108707.54 ± 10507.66	52577.59 ± 6272.43	65550.73 ± 9391.27	44597.45 ± 3281.29
TRA	171.76 ± 10.97	185.30 ± 40.15	333.99 ± 24.57	284.44 ± 6.92	387.55 ± 79.27	291.77 ± 6.29	410.24 ± 61.73	305.22 ± 36.87	209.79 ± 36.74	433.81 ± 27.30	376.04 ± 21.02	401.10 ± 2.14
ANT	3.61 ± 0.38	1.60 ± 0.33	1.64 ± 0.24	0.98 ± 0.31	2.66 ± 0.14	1.25 ± 0.01	3.41 ± 0.62	1.43 ± 0.00	0.81 ± 0.01	4.29 ± 0.55	1.71 ± 0.06	1.66 ± 0.44
IAA	11.08 ± 0.17	14.47 ± 1.90	10.37 ± 1.35	11.81 ± 0.45	15.09 ± 3.83	9.11 ± 0.34	9.92 ± 1.15	9.06 ± 0.13	12.83 ± 0.76	7.24 ± 1.39	7.26 ± 0.77	8.07 ± 0.67
oxIAA	11.48 ± 0.32	10.32 ± 0.05	7.56 ± 0.88	9.23 ± 0.45	12.08 ± 0.21	10.35 ± 1.68	7.70 ± 2.14	8.15 ± 0.86	7.31 ± 0.77	3.91 ± 1.02	4.66 ± 0.42	4.26 ± 0.81
IAAsp	1.26 ± 0.22	1.34 ± 0.20	1.42 ± 0.17	2.55 ± 0.63	1.28 ± 0.18	1.52 ± 0.01	2.25 ± 0.24	0.96 ± 0.12	1.71 ± 0.32	2.66 ± 0.41	2.77 ± 0.05	1.76 ± 0.12

Table S21 Gibberelins content in roots of infected control flax plants and plants treated with Spd (10 mM and 100 mM) and infected with *F. oxysporum*

**GAs [pmol/g FW]**	**2d**			**3d**			**7d**			**14d**		
	**Foln**	**10**	**100**	**Foln**	**10**	**100**	**Foln**	**10**	**100**	**Foln**	**10**	**100**
GA_4	0.01 ± 0.01	0.02 ± 0.01	0.02 ± 0.00	0.02 ± 0.00	0.03 ± 0.00	0.02 ± 0.01	0.04 ± 0.01	0.03 ± 0.00	0.02 ± 0.01	0.02 ± 0.01	0.01 ± 0.00	0.02 ± 0.00
GA_{34}	0.05 ± 0.01	0.03 ± 0.00	0.05 ± 0.00	0.08 ± 0.01	0.09 ± 0.01	0.05 ± 0.01	0.08 ± 0.01	0.06 ± 0.01	0.05 ± 0.01	0.07 ± 0.00	0.04 ± 0.00	0.05 ± 0.01
GA_{53}	0.01 ± 0.00	0.04 ± 0.00	0.02 ± 0.00	0.02 ± 0.00	0.02 ± 0.01	0.04 ± 0.00	0.02 ± 0.00	0.02 ± 0.00	0.02 ± 0.00	0.06 ± 0.01	0.04 ± 0.00	0.05 ± 0.00
GA_{44}	0.04 ± 0.00	0.03 ± 0.00	0.03 ± 0.01	0.04 ± 0.01	0.08 ± 0.01	0.12 ± 0.01	0.15 ± 0.01	0.25 ± 0.03	0.35 ± 0.05	3.89 ± 0.03	0.15 ± 0.03	0.46 ± 0.06
GA_{19}	0.11 ± 0.01	0.12 ± 0.01	0.12 ± 0.01	0.11 ± 0.02	0.12 ± 0.01	0.11 ± 0.01	0.14 ± 0.01	0.14 ± 0.02	0.17 ± 0.01	0.24 ± 0.01	0.20 ± 0.01	0.31 ± 0.01
GA_{20}	0.17 ± 0.02	0.11 ± 0.01	0.07 ± 0.01	0.16 ± 0.01	0.12 ± 0.01	0.11 ± 0.00	0.21 ± 0.02	0.24 ± 0.02	0.17 ± 0.03	1.81 ± 0.21	1.44 ± 0.08	3.52 ± 0.30
GA_1	0.03 ± 0.00	0.02 ± 0.00	0.01 ± 0.00	0.03 ± 0.01	0.03 ± 0.01	0.07 ± 0.01	0.05 ± 0.00	0.03 ± 0.01	0.06 ± 0.01	0.09 ± 0.01	0.08 ± 0.01	0.10 ± 0.01
GA_{29}	0.17 ± 0.02	0.12 ± 0.02	0.22 ± 0.03	0.25 ± 0.02	0.30 ± 0.01	0.16 ± 0.02	0.29 ± 0.05	0.31 ± 0.03	0.67 ± 0.06	0.43 ± 0.05	0.26 ± 0.04	0.39 ± 0.06
GA_8	2.04 ± 0.03	2.78 ± 0.13	2.09 ± 0.11	1.89 ± 0.24	1.97 ± 0.14	1.97 ± 0.17	1.93 ± 0.06	2.22 ± 0.05	2.17 ± 0.10	2.01 ± 0.14	1.87 ± 0.07	1.69 ± 0.05
GA_3	0.11 ± 0.01	0.11 ± 0.02	0.09 ± 0.01	0.16 ± 0.00	0.15 ± 0.01	0.23 ± 0.01	0.17 ± 0.02	0.18 ± 0.02	0.16 ± 0.00	0.12 ± 0.01	0.09 ± 0.01	0.10 ± 0.02

Table S22 Gibberelins content in shoots of infected control flax plants and plants treated with Spd (10 mM and 100 mM) and infected with *F. oxysporum*

GAs [pmol/g FW]	2d			3d			7d			14d		
	Foln	10	100	Foln	10	100	Foln	10	100	Foln	10	100
GA_4	0.03 ± 0.01	0.02 ± 0.01	0.03 ± 0.01	0.02 ± 0.01	0.03 ± 0.00	0.02 ± 0.01	0.03 ± 0.01	0.03 ± 0.01	0.05 ± 0.01	0.02 ± 0.00	0.02 ± 0.00	0.02 ± 0.01
GA_{34}	0.01 ± 0.00	0.01 ± 0.00	0.01 ± 0.00	0.01 ± 0.00	0.01 ± 0.00	0.01 ± 0.00	0.01 ± 0.00	0.01 ± 0.00	0.01 ± 0.00	0.01 ± 0.00	0.01 ± 0.00	0.01 ± 0.00
GA_{53}	0.29 ± 0.04	1.53 ± 0.14	0.08 ± 0.01	0.01 ± 0.01	0.02 ± 0.01	0.02 ± 0.00	0.33 ± 0.04	0.02 ± 0.01	0.03 ± 0.01	0.58 ± 0.06	0.59 ± 0.10	0.31 ± 0.04
GA_{44}	0.03 ± 0.01	0.05 ± 0.01	0.03 ± 0.01	0.05 ± 0.01	0.04 ± 0.01	0.03 ± 0.01	0.06 ± 0.01	0.06 ± 0.01	0.06 ± 0.01	0.09 ± 0.01	0.06 ± 0.02	0.07 ± 0.02
GA_{19}	0.29 ± 0.02	0.30 ± 0.01	0.26 ± 0.01	0.28 ± 0.01	0.28 ± 0.02	0.23 ± 0.01	0.33 ± 0.00	0.25 ± 0.01	0.39 ± 0.01	0.42 ± 0.01	0.45 ± 0.01	0.54 ± 0.03
GA_{20}	0.16 ± 0.01	0.15 ± 0.01	0.11 ± 0.01	0.09 ± 0.01	0.14 ± 0.02	0.12 ± 0.02	0.07 ± 0.01	0.04 ± 0.01	0.14 ± 0.00	0.11 ± 0.01	0.07 ± 0.01	0.08 ± 0.02
GA_1	0.16 ± 0.03	0.24 ± 0.03	0.21 ± 0.02	0.18 ± 0.02	0.24 ± 0.01	0.17 ± 0.01	0.06 ± 0.01	0.06 ± 0.02	0.08 ± 0.01	0.06 ± 0.01	0.06 ± 0.02	0.06 ± 0.01
GA_{29}	0.04 ± 0.01	0.03 ± 0.01	0.03 ± 0.01	0.05 ± 0.01	0.05 ± 0.01	0.03 ± 0.01	0.04 ± 0.01	0.06 ± 0.01	0.08 ± 0.01	0.12 ± 0.01	0.09 ± 0.01	0.06 ± 0.01
GA_8	0.91 ± 0.01	0.85 ± 0.01	0.51 ± 0.06	0.65 ± 0.07	0.82 ± 0.06	0.68 ± 0.04	0.95 ± 0.05	0.95 ± 0.06	1.18 ± 0.03	1.20 ± 0.02	0.74 ± 0.04	1.15 ± 0.11
GA_3	0.31 ± 0.02	0.47 ± 0.01	0.14 ± 0.01	0.11 ± 0.01	0.12 ± 0.02	0.10 ± 0.01	0.19 ± 0.01	0.22 ± 0.02	0.21 ± 0.03	0.25 ± 0.01	0.16 ± 0.03	0.23 ± 0.02

Table S23 Jasmonates, salicylic acid and abscisic content in roots of infected control flax plants and plants treated with Spd (10 mM and 100 mM) and infected with *F. oxysporum*

**JAs + ABA [pmol/g FW]**	**2d**			**3d**			**7d**			**14d**		
	**Foln**	**10**	**100**	**Foln**	**10**	**100**	**Foln**	**10**	**100**	**Foln**	**10**	**100**
JA	0.07 ± 0.01	0.19 ± 0.01	0.07 ± 0.01	0.04 ± 0.02	0.03 ± 0.01	0.02 ± 0.01	0.04 ± 0.00	0.03 ± 0.01	0.05 ± 0.01	0.19 ± 0.04	0.25 ± 0.01	0.15 ± 0.01
9.10-DHJA	0.31 ± 0.17	0.29 ± 0.21	0.06 ± 0.00	0.06 ± 0.02	0.09 ± 0.03	0.05 ± 0.01	0.04 ± 0.02	0.07 ± 0.03	nd.	0.14 ± 0.06	0.16 ± 0.06	0.17 ± 0.08
SA	9.63 ± 1.44	11.85 ± 4.10	6.17 ± 2.17	3.18 ± 0.13	3.92 ± 0.66	7.20 ± 0.75	9.43 ± 0.35	11.05 ± 1.20	20.55 ± 2.73	17.66 ± 1.62	28.00 ± 5.20	49.61 ± 2.93
*cis*OPDA	1814.15 ± 59.46	1913.44 ± 146.28	634.11 ± 89.08	1086.80 ± 84.40	561.42 ± 201.45	699.49 ± 109.07	1963.90 ± 185.62	1878.65 ± 302.79	679.48 ± 183.48	1003.72 ± 162.85	980.75 ± 268.50	708.39 ± 407.12
ABA	0.24 ± 0.00	0.41 ± 0.05	0.36 ± 0.02	0.33 ± 0.04	0.28 ± 0.09	0.42 ± 0.11	0.38 ± 0.05	0.46 ± 0.02	0.20 ± 0.06	0.48 ± 0.09	0.32 ± 0.07	0.49 ± 0.05

nd. not detected

Table S24 Jasmonates, salicylic acid and abscisic content in shoots of infected control flax plants and plants treated with Spd (10 mM and 100 mM) and infected with *F. oxysporum*

JAs + ABA [pmol/g FW]	2d			3d			7d			14d		
	**Foln**	**10**	**100**	**Foln**	**10**	**100**	**Foln**	**10**	**100**	**Foln**	**10**	**100**
JA-Ile	nd.	nd.	nd.	nd.	nd.	nd.	0.17 ± 0.00	0.19 ± 0.02	0.16 ± 0.01	0.96 ± 0.02	1.32 ± 0.02	0.83 ± 0.18
JA	0.09 ± 0.02	0.06 ± 0.02	0.17 ± 0.01	0.18 ± 0.02	0.17 ± 0.02	0.18 ± 0.03	0.05 ± 0.02	0.04 ± 0.01	0.05 ± 0.01	0.09 ± 0.00	0.21 ± 0.02	0.11 ± 0.01
9.10-DHJA	0.28 ± 0.01	0.19 ± 0.09	0.07 ± 0.02	0.09 ± 0.02	0.13 ± 0.02	0.06 ± 0.01	0.07 ± 0.02	0.11 ± 0.01	0.14 ± 0.03	nd.	nd.	nd.
12-OH-JA	6.38 ± 1.68	10.67 ± 0.82	1.30 ± 0.00	2.40 ± 0.01	1.30 ± 0.00	4.11 ± 1.12	6.27 ± 0.68	4.44 ± 0.48	12.88 ± 2.05	1.30 ± 0.00	3.52 ± 0.25	17.41 ± 1.88
SA	2.36 ± 0.13	3.16 ± 0.52	6.87 ± 2.01	1.07 ± 0.38	2.32 ± 0.42	1.46 ± 0.07	2.26 ± 0.00	1.52 ± 0.70	1.60 ± 0.47	3.04 ± 0.42	5.89 ± 0.40	3.23 ± 0.27
*cis*OPDA	1873.39 ± 41.42	1553.53 ± 276.60	1868.21 ± 121.01	1906.53 ± 420.23	3402.91 ± 340.88	2389.97 ± 91.42	1892.36 ± 67.52	1628.30 ± 73.79	742.97 ± 99.90	2173.19 ± 69.60	1570.90 ± 145.23	993.42 ± 416.63
ABA	0.93 ± 0.11	0.87 ± 0.03	1.65 ± 0.07	0.66 ± 0.13	1.39 ± 0.09	1.58 ± 0.18	0.93 ± 0.11	1.42 ± 0.29	1.29 ± 0.04	1.10 ± 0.02	0.95 ± 0.10	1.24 ± 0.13

nd. not detected
